# Association of Circadian Syndrome with Incident Risk of 23 Site-Specific Cancers: A Large-Scale Prospective Study in the UK Biobank

**DOI:** 10.34133/research.1426

**Published:** 2026-09-22

**Authors:** Jie Wang, Feipeng Jiang, Ruicheng Wu, Dilinaer Wusiman, Koo Han Yoo, Leibo Wang, Zhouting Tuo, Zhaojie Lyu, Qi Zhang, Jianjun Ren, Meixia Zhang, Dechao Feng

**Affiliations:** ^1^ Urology and Nephrology Center, Department of Urology, Zhejiang Provincial People’s Hospital (Affiliated People’s Hospital), Hangzhou Medical College, Hangzhou, Zhejiang 310014, China.; ^2^ Department of Urology, Institute of Urology, West China Hospital, Sichuan University, Chengdu 610041, China.; ^3^ Department of Ophthalmology and Laboratory of Macular Disease, West China Hospital, Sichuan University, Chengdu 610041, Sichuan, China.; ^4^ Division of Surgery and Interventional Science, University College London, London W1W 7TS, UK.; ^5^ Purdue Institute for Cancer Research, Purdue University, West Lafayette, IN 47907, USA.; ^6^ Department of Urology, Kyung Hee University, Seoul, South Korea.; ^7^ Institute of Human Genetics, Jena University Hospital, Jena, Germany.; ^8^ Department of Urology, Institute of Precision Medicine, Shenzhen Key Laboratory of Male Reproduction and Genetics, Peking University Shenzhen Hospital, Shenzhen 518036, Guangdong, China.; ^9^ Department of Otolaryngology–Head and Neck Surgery, West China Hospital, Sichuan University, Chengdu 610041, Sichuan, China.

## Abstract

Circadian disruption and metabolic dysfunction are linked to chronic disease, but whether circadian syndrome, which combines metabolic, sleep, and mood traits, relates to overall and site-specific cancer risk remains unclear. We prospectively studied 367,832 cancer-free participants in the UK Biobank, of whom 82,547 (22.4%) had circadian syndrome, defined by at least 4 of 7 features: abdominal obesity, high triglycerides, low levels of high-density lipoprotein cholesterol, high blood pressure, impaired glucose regulation, short sleep, and depression. Associations with overall cancer and 23 site-specific cancers were evaluated using Cox proportional-hazards and competing-risk models, with additional dose–response, biomarker, and population-burden analyses. During follow-up, 47,695 participants developed cancer. Circadian syndrome was associated with a higher incidence of overall cancer (hazard ratio [HR], 1.12; 95% confidence interval [CI], 1.10 to 1.14). The strongest positive associations were observed for endometrial cancer (HR, 1.88; 95% CI, 1.71 to 2.08), liver cancer (HR, 1.67; 95% CI, 1.47 to 1.89), and kidney cancer (HR, 1.55; 95% CI, 1.41 to 1.70). Findings remained similar after accounting for death from other causes and across sensitivity analyses, whereas inverse associations for prostate and brain cancers were exploratory. Sex-hormone-binding globulin statistically accounted for 51.0% of the endometrial cancer association, and C-reactive protein accounted for 26.4% of the overall cancer association. The highest population-attributable fractions were estimated for endometrial (13.5%) and liver cancers (13.4%). Circadian syndrome may help identify populations at increased cancer risk and inform prevention strategies integrating circadian and metabolic health.

## Introduction

In modern society, circadian disruption has become increasingly common owing to shift work, light-at-night exposure, and other lifestyle changes [1]. The circadian system is fundamental to physiological homeostasis, coordinating metabolic regulation, immune function, endocrine signaling, and cellular proliferation [2–5]. To capture the broader pathophysiological interplay between circadian disruption and metabolic dysfunction, circadian syndrome (CircS) was formally proposed by Zimmet and colleagues [6] as an expanded construct that extends the traditional metabolic syndrome framework by incorporating short sleep duration and depression in addition to central obesity, elevated blood pressure, impaired glucose regulation, hypertriglyceridemia, and reduced high-density lipoprotein cholesterol. The incremental value of CircS over the traditional metabolic syndrome has been demonstrated in multiple independent cohorts: CircS predicted cardiovascular disease more strongly than the metabolic syndrome in both Chinese adults and the US National Health and Nutrition Examination Survey (NHANES) population [7,8], and CircS—but not the metabolic syndrome—was an important predictor of mild cognitive impairment in the China Health and Retirement Longitudinal Study (CHARLS) cohort [9]. This multidimensional construct may better reflect the integrated disturbance of biological timing, metabolism, and affective regulation than isolated metabolic or sleep-related indicators alone [6,10]. Prior evidence has also shown that traditional metabolic syndrome is associated with increased risk of several common cancers, supporting the relevance of examining a broader circadian-metabolic phenotype in oncologic epidemiology [11].

The biological plausibility linking CircS to carcinogenesis is supported by several interconnected pathways. Circadian and metabolic disruption may promote chronic low-grade inflammation, impair DNA damage repair and apoptosis, alter endocrine and immune regulation, and amplify insulin- and growth-factor-related proliferative signaling [3,4,12–14]. These mechanisms may be particularly relevant for obesity-related, hormone-dependent, and hepatometabolic malignancies, although their phenotypic consequences may differ across tissues because malignant evolution is also shaped by phenotypic plasticity and microenvironmental regulation [15–18]. More broadly, cancer development and progression are shaped by immune-evasion mechanisms as well as reciprocal interactions among tumor cells, host immunity, and the tumor-associated microbiome [19–21]. Recent work has begun to establish the clinical relevance of CircS beyond cardiovascular disease. In a dual-cohort analysis of NHANES and the UK Biobank, CircS predicted all-cause and cause-specific mortality—including cancer mortality—more strongly than the metabolic syndrome, indicating that the circadian components add prognostic information beyond traditional metabolic risk clustering [22]. In a Chinese middle-aged and elderly population, CircS was associated with cancer risk, an association partly mediated by the triglyceride-glucose index, a surrogate of insulin resistance [23]. However, these studies evaluated mortality or composite cancer outcomes rather than the full spectrum of site-specific cancer incidence. Accordingly, prospective evidence evaluating CircS in relation to overall and site-specific cancer incidence remains limited [6,24]. Beyond cardiovascular and metabolic outcomes, CircS has been associated with cardio-kidney events and all-cause mortality in the UK Biobank [25], with dose-dependent increases in all-cause mortality in the CHARLS and NHANES cohorts [26], and with chronic kidney disease [27], nonalcoholic fatty liver disease [28], and multimorbidity [29]. To our knowledge, Bai et al. [23] remains the only prior study of CircS and cancer incidence, and it was conducted in a Chinese population.

In middle-aged and older populations, noncancer death may act as a competing event and lead to overestimation of cumulative incidence when not properly accounted for [30]. Evidence is also limited regarding whether CircS-related associations differ across organ systems, exposure severity, and population subgroups, and the population-level cancer burden attributable to CircS has not been well quantified. Therefore, using the UK Biobank, a large population-based prospective cohort [31,32], we aimed to examine the associations of baseline CircS, modeled as both a binary exposure and a continuous severity index, with incident overall cancer and 23 site-specific cancers. We further assessed competing-risk associations, dose–response patterns, candidate biomarker mediation, and population-attributable fractions to clarify the epidemiological and public health relevance of CircS for cancer prevention.

## Results

### Study population and baseline characteristics

Among 501,956 UK Biobank participants, 19,407 with prevalent cancer, 25,367 with missing variables required for CircS construction, and 89,350 with missing key covariates were excluded, leaving 367,832 participants in the final analytic cohort. For overall cancer and each site-specific outcome, Table S1 reports the number of participants at risk, incident cases, accrued person-years, and crude incidence rates, overall and according to CircS status. All exposure metrics used the same outcome-specific analytic populations reported in Table S1. During follow-up, 47,695 incident overall cancer events were documented. Of the included participants, 82,547 (22.4%) met the binary CircS definition and 285,285 (77.6%) did not. Among participants with CircS, short sleep was present in 9,298 (11.3%) and depression in 27,495 (33.3%); the 15 most common exact component combinations accounted for 88.2% (Fig. S1).

**Fig. 1.**
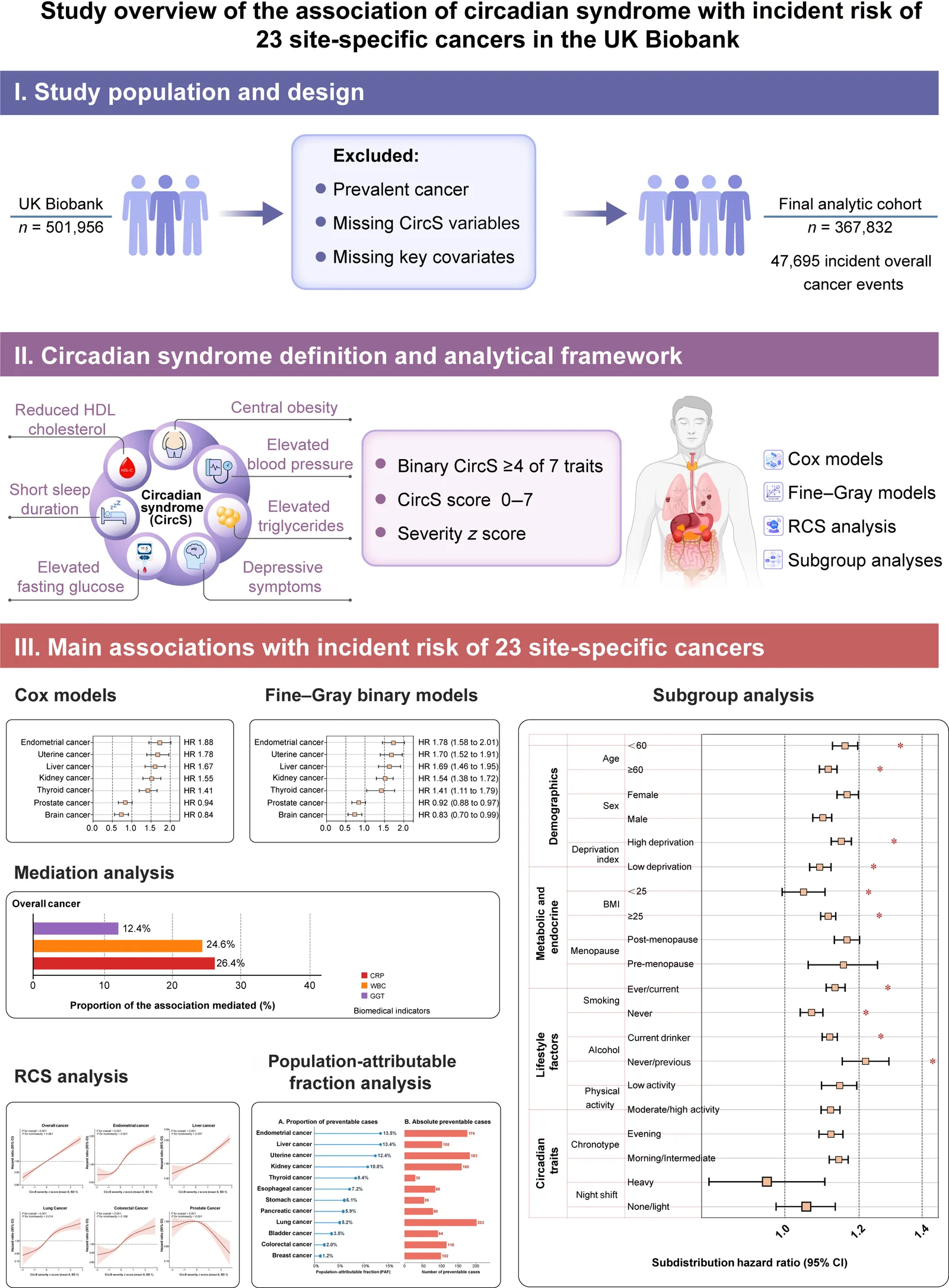
The overview of this study. RCS, restricted cubic spline; BMI, body mass index; CRP, C-reactive protein; WBC, white blood cell count; GGT, gamma-glutamyl transferase; HDL, high-density lipoprotein; HR, hazard ratio. Red asterisks indicate statistically significant interaction terms (*P* for interaction < 0.05).

Baseline characteristics according to binary CircS status are presented in Table 1. Compared with participants without CircS, those with CircS were older (mean [SD] age, 58.80 [7.37] vs. 55.30 [8.16] y), were more likely to be male (54% vs. 47%), and had less favorable socioeconomic profiles, including higher Townsend deprivation index values and a lower proportion with college or university education. They also had higher prevalences of current smoking and low physical activity. As expected given the syndrome definition, participants with CircS had a more adverse cardiometabolic profile, including higher waist circumference, body mass index (BMI), blood pressure, triglycerides, fasting glucose, glycated hemoglobin, and C-reactive protein (CRP) levels, together with lower high-density lipoprotein cholesterol (HDL-C) levels. Short sleep duration and depression were also more common in the CircS group. Figure 1 illustrates the study overview.

**Table 1.** Baseline characteristics of the study population by circadian syndrome status. Values are presented as mean (SD), median [Q1, Q3], or *n* (%) unless otherwise indicated. *P* values compare participants without CircS (score 0 to 3) and those with CircS (score ≥4). Continuous variables were compared using the Wilcoxon rank-sum test and categorical variables using the Pearson *χ*^2^ test.

Characteristic	Overall *N* = 367,832	No CircS (score 0–3) *N* = 285,285	CircS (score ≥4) *N* = 82,547	*P* value
Age, y, mean (SD)	56.08 (8.12)	55.30 (8.16)	58.80 (7.37)	<0.001
Female, *n* (%)	190,777 (52%)	152,503 (53%)	38,274 (46%)	<0.001
White, *n* (%)	349,681 (95%)	271,991 (95%)	77,690 (94%)	<0.001
Townsend deprivation index, median [Q1, Q3]	−2.24 [−3.70, 0.32]	−2.34 [−3.75, 0.11]	−1.85 [−3.48, 1.04]	<0.001
Education, *n* (%)				<0.001
College/university	135,726 (37%)	112,357 (40%)	23,369 (29%)	
A/AS levels	122,212 (33%)	93,961 (33%)	28,251 (34%)	
O/GCSE levels	58,414 (16%)	44,922 (16%)	13,492 (16%)	
Other/none	49,412 (14%)	32,595 (11%)	16,817 (21%)	
Employed, *n* (%)	352,593 (95.9%)	273,018 (95.7%)	79,575 (96.4%)	<0.001
Smoking, *n* (%)				<0.001
Never	202,601 (55%)	164,083 (58%)	38,518 (47%)	
Previous	127,983 (35%)	93,768 (33%)	34,215 (41%)	
Current	37,248 (10%)	27,434 (9.6%)	9,814 (12%)	
Alcohol, *n* (%)				<0.001
Never	14,065 (3.8%)	9,674 (3.4%)	4,391 (5.3%)	
Previous	12,270 (3.3%)	7,914 (2.8%)	4,356 (5.3%)	
Current	341,497 (93%)	267,697 (94%)	73,800 (89%)	
Physical activity, *n* (%)				<0.001
Low	67,973 (18%)	47,305 (17%)	20,668 (25%)	
Moderate	149,402 (41%)	115,599 (41%)	33,803 (41%)	
High	150,457 (41%)	122,381 (43%)	28,076 (34%)	
Sleep duration, h/d, mean (SD)	7.16 (1.07)	7.19 (0.98)	7.07 (1.32)	<0.001
Waist circumference, cm, mean (SD)	90.11 (13.37)	86.96 (11.82)	100.98 (12.68)	<0.001
BMI, kg/m^2^, mean (SD)	27.27 (4.69)	26.21 (3.98)	30.96 (5.06)	<0.001
Systolic blood pressure, mm Hg, mean (SD)	137.37 (18.54)	135.70 (18.59)	143.13 (17.16)	<0.001
Diastolic blood pressure, mm Hg, mean (SD)	82.18 (10.16)	81.46 (10.11)	84.67 (9.91)	<0.001
Triglycerides, mmol/L, median [Q1, Q3]	1.47 [1.04, 2.13]	1.34 [0.97, 1.89]	2.05 [1.48, 2.82]	<0.001
HDL-C, mmol/L, mean (SD)	1.45 (0.38)	1.51 (0.38)	1.24 (0.32)	<0.001
Fasting glucose, mmol/L, mean (SD)	5.10 (1.21)	4.94 (0.76)	5.64 (2.00)	<0.001
HbA1c, mmol/mol, mean (SD)	35.89 (6.50)	34.47 (4.10)	40.71 (9.95)	<0.001
C-reactive protein, mg/L, median [Q1, Q3]	1.26 [0.63, 2.60]	1.10 [0.56, 2.22]	2.03 [1.04, 3.99]	<0.001
Central obesity, *n* (%)	116,989 (32%)	55,897 (20%)	61,092 (74%)	<0.001
Elevated blood pressure, *n* (%)	261,715 (71%)	182,764 (64%)	78,951 (96%)	<0.001
Elevated triglycerides, *n* (%)	184,896 (50%)	106,000 (37%)	78,896 (96%)	<0.001
Reduced HDL-C, *n* (%)	129,434 (35%)	58,471 (20%)	70,963 (86%)	<0.001
Impaired glucose regulation, *n* (%)	64,197 (17%)	21,442 (7.5%)	42,755 (52%)	<0.001
Short sleep duration, *n* (%)	18,184 (4.9%)	8,886 (3.1%)	9,298 (11%)	<0.001
Depression, *n* (%)	64,286 (17%)	36,791 (13%)	27,495 (33%)	<0.001
Continuous severity *z* score, mean (SD)	0.00 (1.00)	−0.30 (0.80)	1.03 (0.95)	<0.001

BMI, body mass index; CircS, circadian syndrome; HbA1c, glycated hemoglobin; HDL-C, high-density lipoprotein cholesterol; Q1, first quartile; Q3, third quartile; A/AS levels, General Certificate of Education Advanced Level or Advanced Subsidiary Level; O/GCSE levels, General Certificate of Education Ordinary Level or General Certificate of Secondary Education

### Circadian syndrome and incident cancer in Cox models

**Fig. 2.**
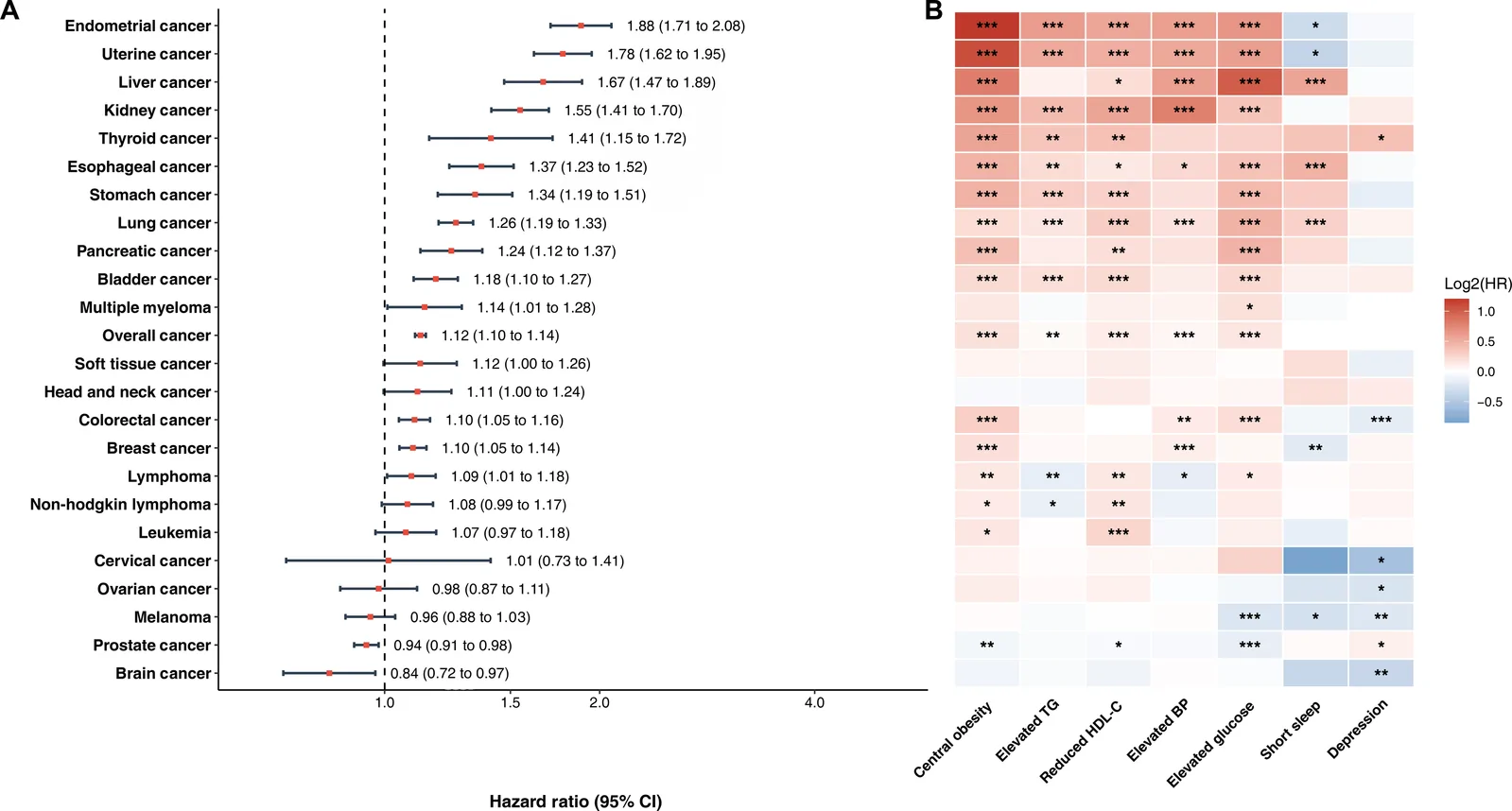
Associations of binary circadian syndrome (CircS) status and individual CircS components with overall and site-specific incident cancer risk. (A) HRs and 95% confidence intervals (CIs) for the associations of binary CircS status (CircS score ≥4 vs. 0 to 3) with overall and site-specific incident cancers in fully adjusted Cox proportional-hazards models. The dashed vertical line indicates the null value (HR = 1.0). (B) The corresponding associations of individual CircS components with overall and site-specific cancer outcomes in fully adjusted Cox models, displayed as log2(HR). Warmer colors indicate positive associations and cooler colors indicate inverse associations. CircS components included central obesity, elevated triglycerides (TG), reduced high-density lipoprotein cholesterol (HDL-C), elevated blood pressure (BP), elevated glucose, short sleep, and depression. *, **, and *** indicate *P* < 0.05, *P* < 0.01, and *P* < 0.001, respectively. *P* values for the site-specific analyses were adjusted for multiple testing using the Benjamini–Hochberg procedure.

In the fully adjusted Cox models, binary CircS was associated with a higher risk of overall incident cancer (hazard ratio [HR], 1.12; 95% confidence interval [CI], 1.10 to 1.14; Fig. 2). Among the site-specific cancers, the largest positive HR was observed for endometrial cancer (HR, 1.88; 95% CI, 1.71 to 2.08), the most specific uterine outcome; the broader uterine cancer category, which includes endometrial cancer, showed a consistent association (HR, 1.78; 95% CI, 1.62 to 1.95), and these nested outcomes reflect a single underlying association. Large positive HRs were also observed for liver cancer (HR, 1.67; 95% CI, 1.47 to 1.89), kidney cancer (HR, 1.55; 95% CI, 1.41 to 1.70), and thyroid cancer (HR, 1.41; 95% CI, 1.15 to 1.72). Positive associations were also observed for esophageal, stomach, lung, pancreatic, bladder, colorectal, breast, lymphoma, and multiple myeloma outcomes. Associations for head and neck cancer and soft tissue cancer were positive but more borderline. By contrast, inverse associations were observed for brain cancer (HR, 0.84; 95% CI, 0.72 to 0.97) and prostate cancer (HR, 0.94; 95% CI, 0.91 to 0.98), whereas estimates for melanoma, ovarian cancer, cervical cancer, leukemia, and non-Hodgkin lymphoma were closer to the null.

When CircS was modeled as a continuous score or as a standardized severity *z* score, the overall pattern for overall cancer and several positively associated site-specific cancers remained broadly similar (Figs. S2 and S3). For overall cancer, the corresponding HRs were 1.04 (95% CI, 1.03 to 1.04) per 1-point higher CircS score and 1.02 (95% CI, 1.02 to 1.03) per 1-SD increase in the CircS severity *z* score. The inverse association for prostate cancer was retained in both continuous analyses, whereas the estimate for brain cancer attenuated toward the null.

### Associations of circadian syndrome with incident cancer in Fine–Gray models

**Fig. 3.**
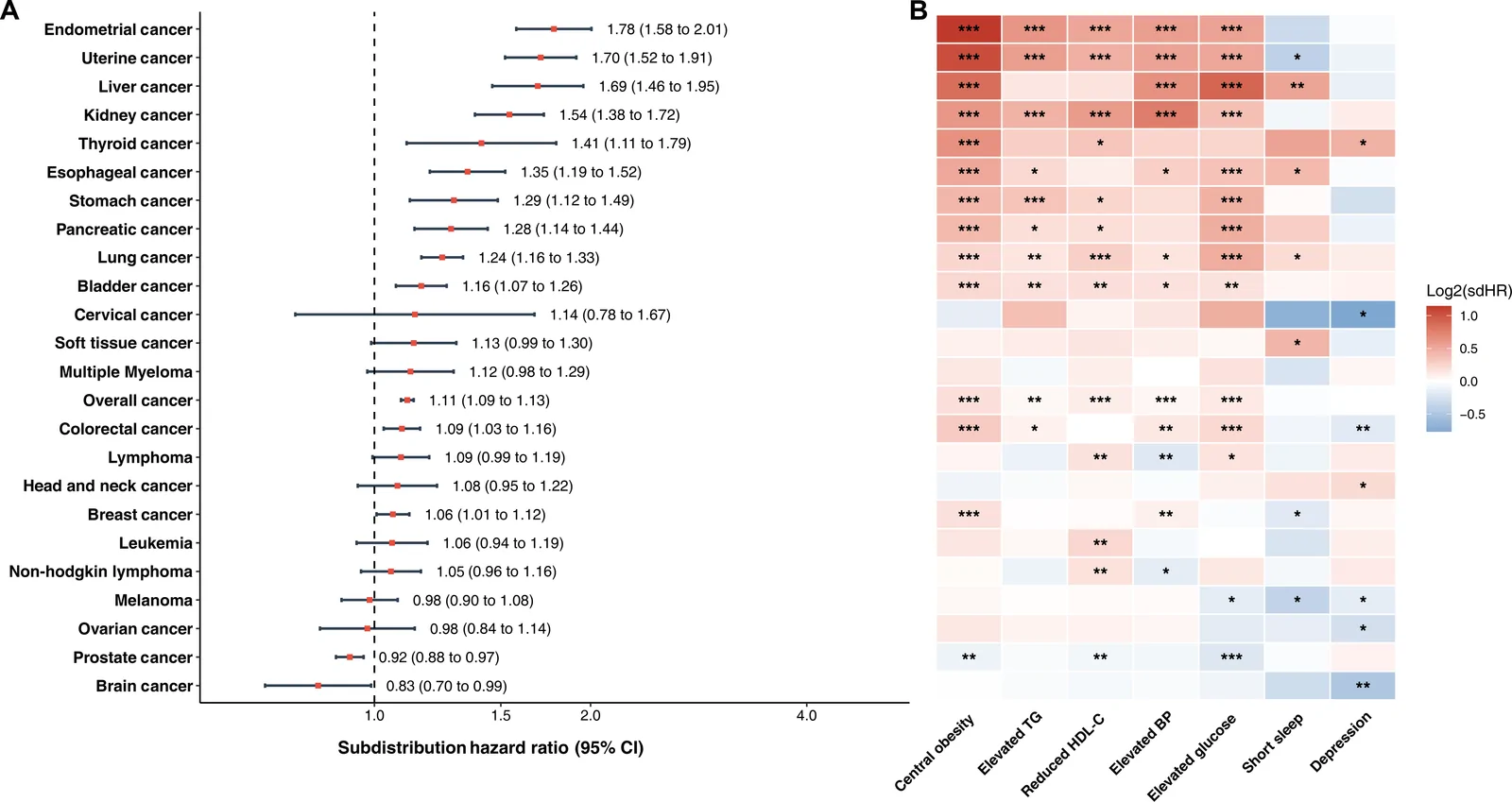
Associations of binary CircS status and individual CircS components with overall and site-specific incident cancer in Fine–Gray models. (A) Subdistribution hazard ratios (sdHRs) and 95% CIs for the associations of binary circadian syndrome (CircS) status (CircS score ≥4 vs. 0 to 3) with overall and site-specific incident cancers in fully adjusted Fine–Gray models, with noncancer death treated as the competing event. The dashed vertical line indicates the null value (sdHR = 1.0). (B) The corresponding associations of individual CircS components with overall and site-specific cancer outcomes in fully adjusted Fine–Gray models, displayed as log2(sdHR). Warmer colors indicate positive associations and cooler colors indicate inverse associations. CircS components included central obesity, elevated TG, reduced HDL-C, elevated BP, elevated glucose, short sleep, and depression. *, **, and *** indicate *P* < 0.05, *P* < 0.01, and *P* < 0.001, respectively. *P* values for the site-specific analyses were adjusted for multiple testing using the Benjamini–Hochberg procedure.

In the fully adjusted Fine–Gray models, binary CircS was associated with a higher subdistribution hazard of overall incident cancer (subdistribution hazard ratio [sdHR], 1.11; 95% CI, 1.09 to 1.13; Fig. 3). The strongest positive association was observed for endometrial cancer (sdHR, 1.78; 95% CI, 1.58 to 2.01), and the broader uterine cancer category showed a consistent association (sdHR, 1.70; 95% CI, 1.52 to 1.91). Strong associations were also observed for liver cancer (sdHR, 1.69; 95% CI, 1.46 to 1.95) and kidney cancer (sdHR, 1.54; 95% CI, 1.38 to 1.72). Inverse associations were observed for prostate cancer (sdHR, 0.92; 95% CI, 0.88 to 0.97) and brain cancer (sdHR, 0.83; 95% CI, 0.70 to 0.99). When CircS was modeled as a continuous score or standardized severity *z* score, the overall pattern remained broadly similar (Figs. S4 and S5). For overall cancer, the corresponding sdHRs were 1.03 (95% CI, 1.03 to 1.04) per 1-point higher CircS score and 1.02 (95% CI, 1.02 to 1.02) per 1-SD increase in the CircS severity *z* score. The inverse association for prostate cancer was retained in both continuous analyses, whereas the estimate for brain cancer attenuated toward the null.

### Dose–response associations of circadian syndrome severity with incident cancer

**Fig. 4.**
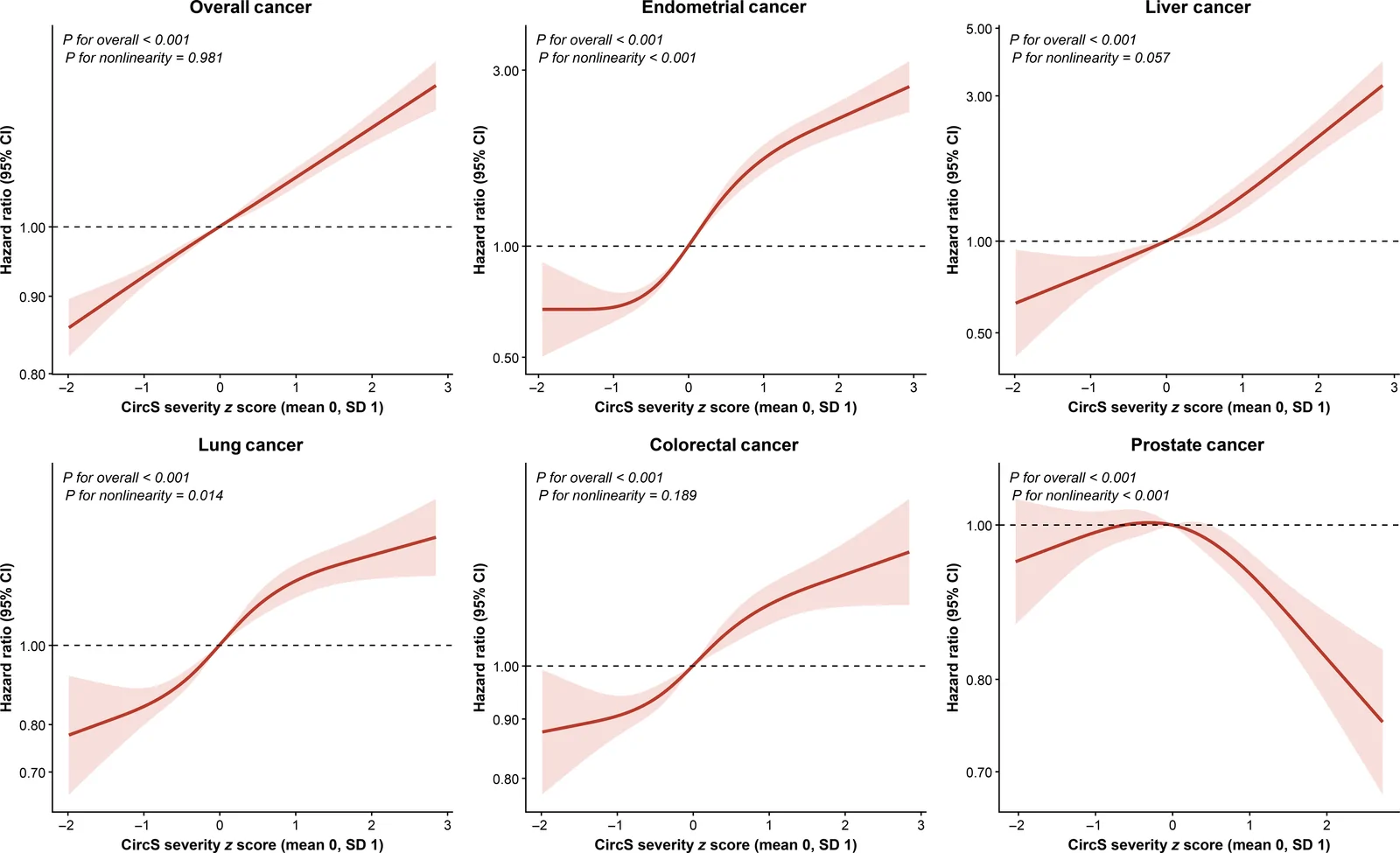
Restricted cubic spline analyses of CircS severity and selected incident cancers. Restricted cubic spline curves showing the associations between the standardized CircS severity *z* score and selected incident cancer outcomes in fully adjusted Cox proportional-hazards models. Representative curves are shown for overall cancer, endometrial cancer, liver cancer, lung cancer, colorectal cancer, and prostate cancer. Knots were placed at the 5th, 35th, 65th, and 95th percentiles of the *z*-score distribution, with the median *z* score used as the reference. Solid lines indicate HRs, and shaded areas indicate 95% CIs. *P* values for the overall association and for nonlinearity are shown in each panel. *P* values for nonlinearity were adjusted for multiple testing using the Benjamini–Hochberg procedure.

Restricted cubic spline analyses showed a predominantly linear association between CircS severity and overall cancer risk (*P* for overall association <0.001; *P* for nonlinearity = 0.981; Fig. 4). A similar near-linear pattern was observed for colorectal cancer (*P* for nonlinearity = 0.189). By contrast, endometrial cancer showed clear evidence of nonlinearity (*P* for nonlinearity < 0.001), with risk increasing more steeply at higher values of the CircS severity *z* score. Lung cancer also showed evidence of nonlinearity (*P* for nonlinearity = 0.014), with risk increasing across the midrange of the *z*-score distribution and appearing to plateau at higher values. Liver cancer showed a strong overall association with only borderline evidence of nonlinearity (*P* for nonlinearity = 0.057), whereas prostate cancer showed an inverse nonlinear pattern (*P* for nonlinearity < 0.001).

In supplementary spline analyses, most additional site-specific outcomes with positive overall associations showed little evidence of nonlinearity. Uterine cancer was a notable exception, with evidence of a nonlinear positive association (*P* for nonlinearity < 0.001), whereas breast, kidney, and bladder cancers showed only borderline evidence of nonlinearity. Additional spline curves are provided in Figs. S6 to S8.

### Subgroup associations of circadian syndrome with selected incident cancers

**Fig. 5.**
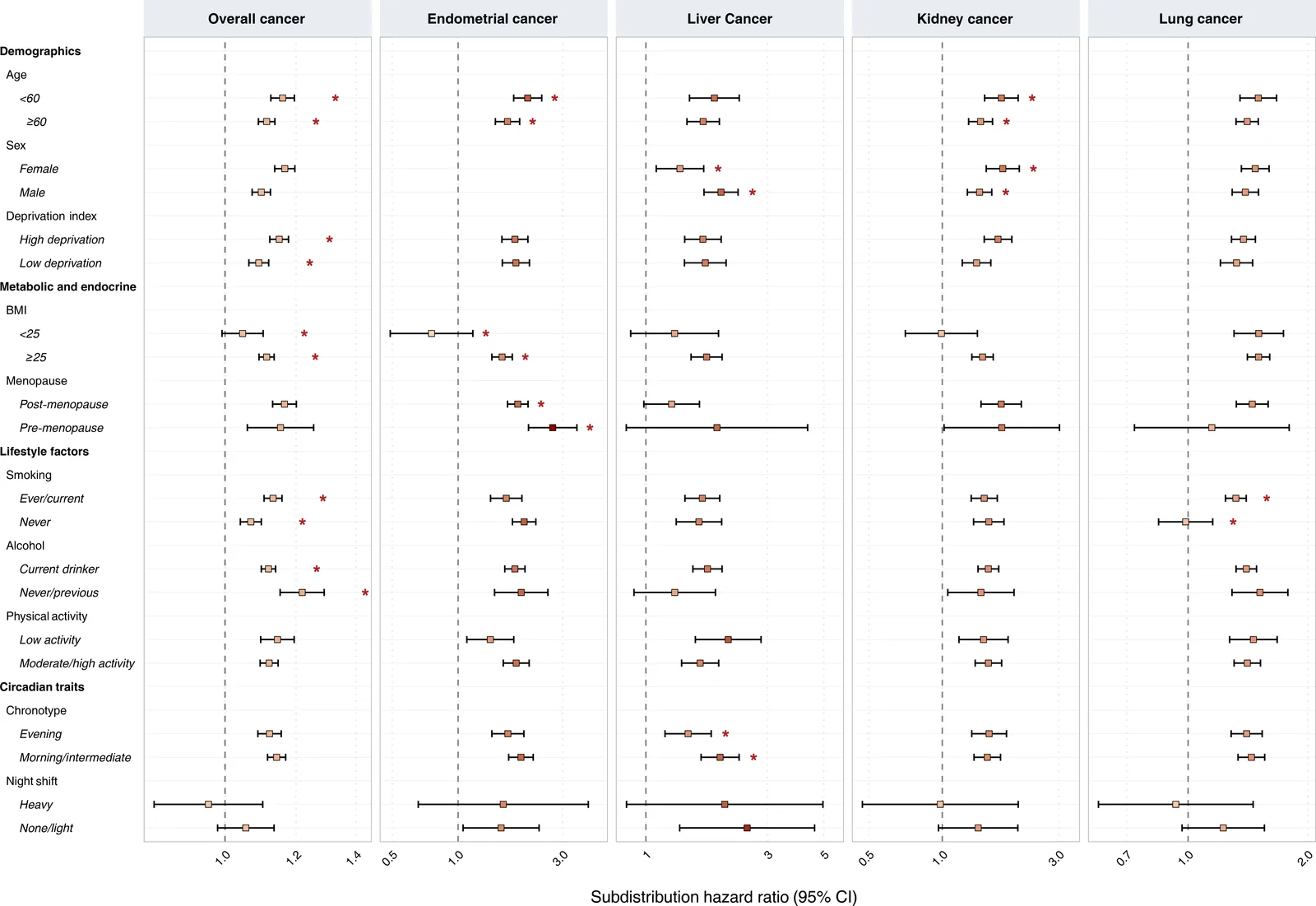
Subgroup associations of circadian syndrome with selected incident cancers in Fine–Gray models. Forest plots showing sdHRs and 95% CIs for the associations of binary CircS status with overall cancer, endometrial cancer, liver cancer, kidney cancer, and lung cancer across prespecified subgroups in fully adjusted Fine–Gray models. Red asterisks indicate significant interaction terms (*P* for interaction < 0.05). *X*-axes are shown on a log scale and vary across panels. *P* values for the interaction tests were adjusted for multiple testing using the Benjamini–Hochberg procedure.

Subgroup analyses are shown in Fig. 5. For overall cancer, the positive association with CircS was generally consistent across prespecified subgroups, although evidence of heterogeneity was observed by age, Townsend deprivation, BMI, smoking, and alcohol use. The association tended to be stronger among participants younger than 60 years, those with higher deprivation, those with BMI of at least 25 kg/m^2^, ever or current smokers, and never or previous drinkers than in the corresponding comparison groups.

Among site-specific outcomes, endometrial cancer showed the most pronounced subgroup heterogeneity. The positive association was stronger among women younger than 60 years than among older women, stronger among women with BMI of at least 25 kg/m^2^ than among those with BMI below 25 kg/m^2^, and differed by menopausal status. Additional heterogeneity was observed by sex for liver and kidney cancer and by smoking status for lung cancer. No clear and consistent heterogeneity was observed across chronotype or night-shift strata. Overall, however, the direction of association remained broadly similar across most subgroup strata. In supplementary analyses, the inverse association between CircS and prostate cancer was generally consistent across prespecified subgroup strata (Fig. S9).

### Sensitivity analyses

Multiple-imputation sensitivity analyses are shown in Figs. S10 and S11. The direction and magnitude of these associations were materially consistent with the complete-case analyses. Associations for lung, endometrial, and liver cancer remained positive after site-targeted covariate adjustment (Table S2). Additional sensitivity analyses are shown in Figs. S12 and S13. After excluding incident cancers occurring within the first 2 years of follow-up, the association between binary CircS and overall incident cancer remained unchanged (HR, 1.12; 95% CI, 1.10 to 1.14). In a model additionally adjusted for BMI and CRP, binary CircS remained associated with overall incident cancer (HR, 1.11; 95% CI, 1.09 to 1.14). The site-specific patterns were materially similar to the primary analyses.

### Candidate biomarker mediation analyses

**Fig. 6.**
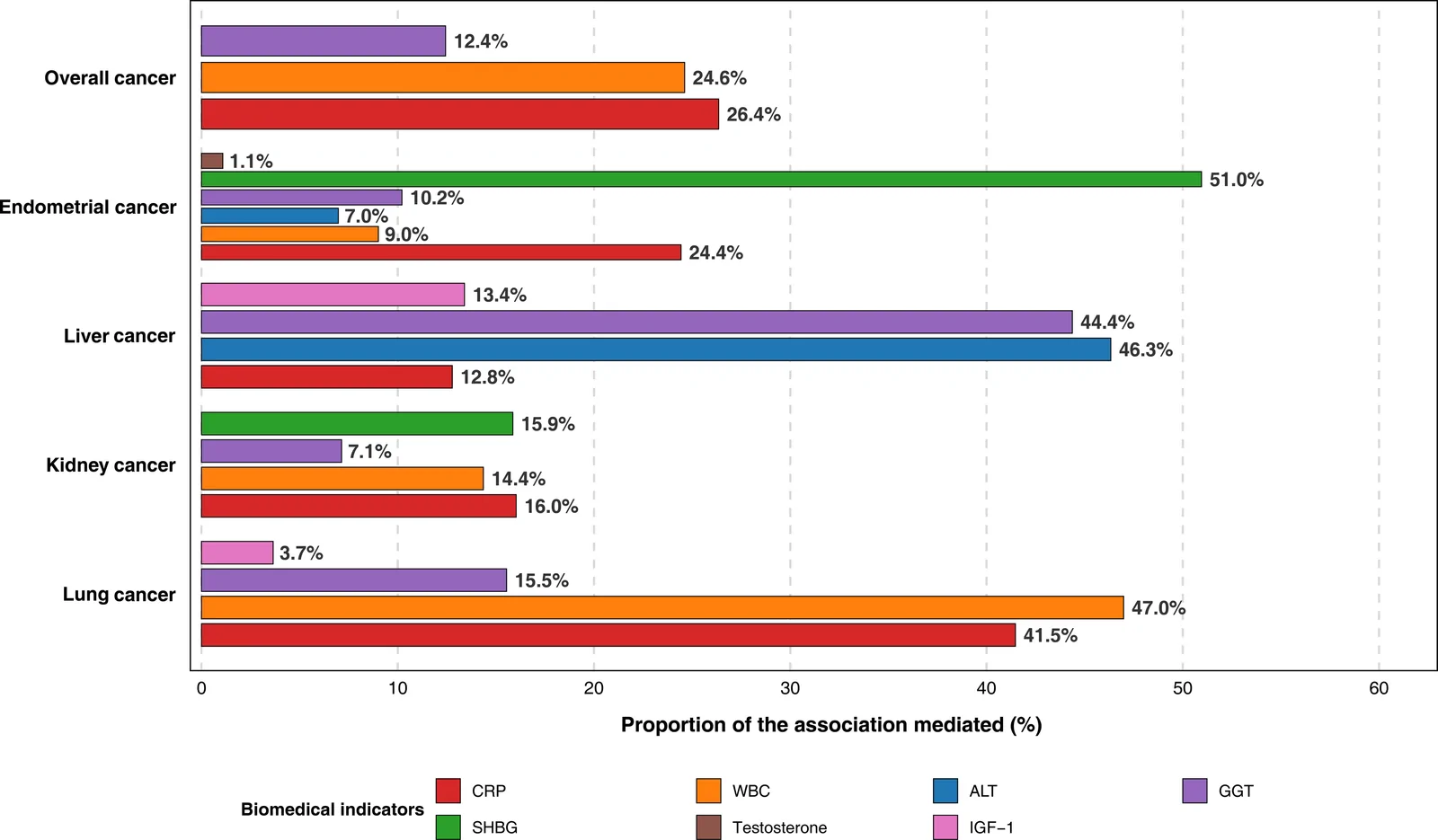
Estimated proportions of the association between circadian syndrome and incident cancer mediated by selected biomarkers. Bar plot showing the estimated proportion of the association between CircS and incident cancer mediated by selected biomarkers for overall cancer, endometrial cancer, liver cancer, kidney cancer, and lung cancer. Only biomarkers with statistically significant indirect effects (*P* < 0.05) are displayed. Biomarkers included C-reactive protein (CRP), white blood cell count (WBC), alanine aminotransferase (ALT), gamma-glutamyl transferase (GGT), sex-hormone-binding globulin (SHBG), testosterone, and insulin-like growth factor 1 (IGF-1). *P* values for the biomarker tests were adjusted for multiple testing using the Benjamini–Hochberg procedure.

Candidate biomarker analyses suggested that several biomarkers were statistically associated with the CircS–cancer relation and may partly reflect underlying biological pathways (Fig. 6 and Table S3). For overall cancer, the largest estimated proportions of association explained were observed for CRP (26.4%), white blood cell count (WBC; 24.6%), and gamma-glutamyl transferase (GGT; 12.4%). For endometrial cancer, sex-hormone-binding globulin (SHBG) showed the largest estimated proportion of association explained (51.0%), followed by CRP (24.4%). For liver cancer, alanine aminotransferase and GGT showed the largest estimated proportions, accounting for 46.3% and 44.4% of the association, respectively. For lung cancer, WBC and CRP showed estimated proportions of 47.0% and 41.5%, respectively. For kidney cancer, estimated proportions were smaller, with CRP, SHBG, and WBC each accounting for approximately 14.0% to 16.0% of the association. Overall, no single biomarker fully explained the observed associations.

### Population-attributable fraction analyses

**Fig. 7.**
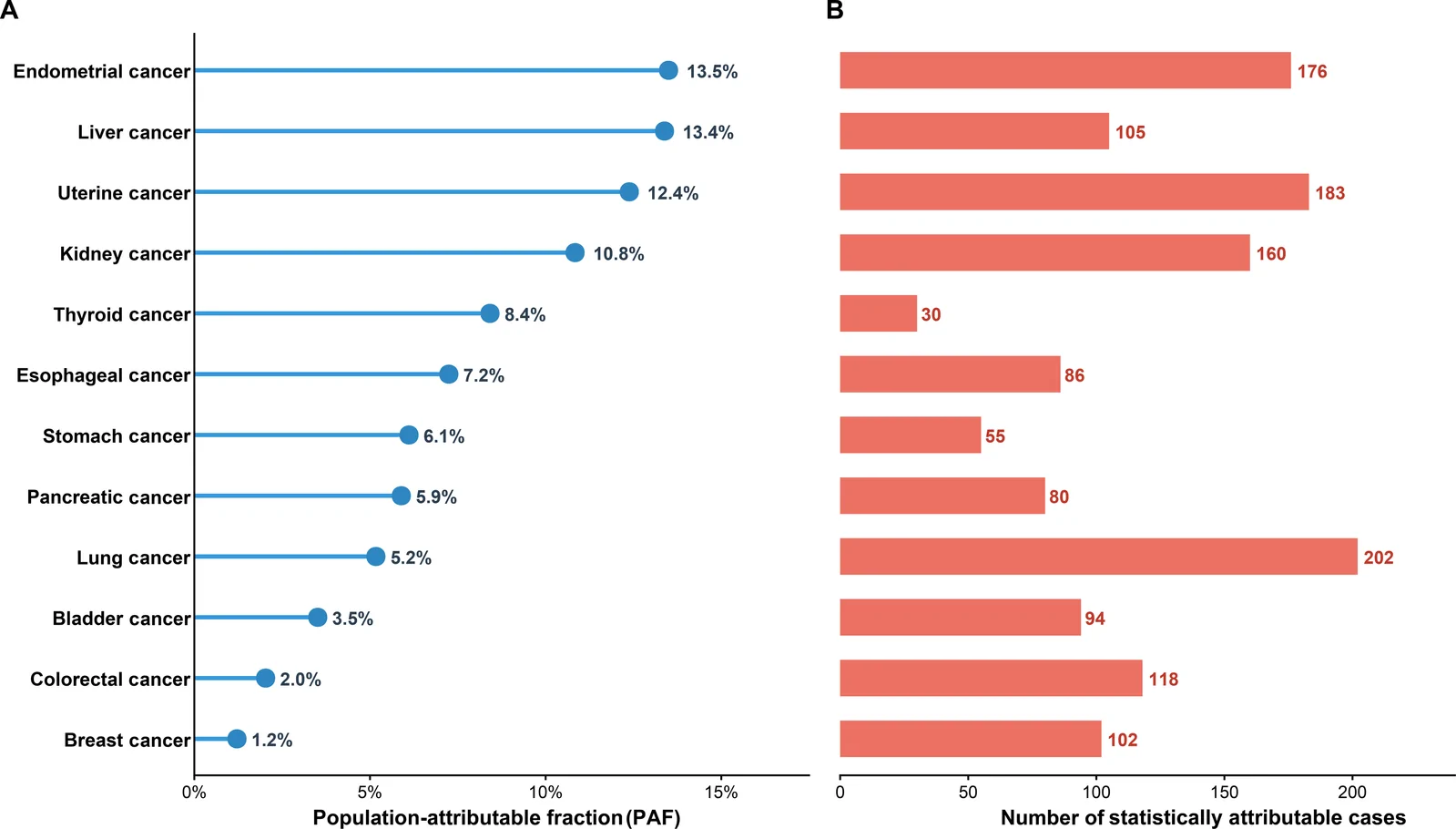
Estimated population-attributable fractions (PAFs) and attributable case counts for selected incident cancers associated with CircS. (A) The estimated PAF for selected site-specific incident cancers associated with CircS, based on the fully adjusted competing-risk model and the observed prevalence of CircS in the study population. (B) The corresponding estimated attributable case counts in the cohort. Cancer sites are ordered by descending PAF and aligned across both panels to facilitate comparison between proportional and absolute burden.

Population-attributable fraction (PAF) analyses suggested a nontrivial population burden statistically attributable to CircS across several site-specific cancers. The highest estimated PAFs were observed for endometrial cancer (13.5%; 95% CI, 10.4% to 16.8%), liver cancer (13.4%; 95% CI, 9.3% to 17.6%), uterine cancer (12.4%; 95% CI, 9.5% to 15.4%), and kidney cancer (10.8%; 95% CI, 7.9% to 13.9%), followed by thyroid cancer (8.4%; 95% CI, 2.4% to 15.1%), esophageal cancer (7.2%; 95% CI, 4.2% to 10.5%), stomach cancer (6.1%; 95% CI, 2.6% to 9.8%), pancreatic cancer (5.9%; 95% CI, 3.0% to 9.0%), and lung cancer (5.2%; 95% CI, 3.5% to 6.9%). Lower PAF estimates were observed for bladder cancer (3.5%; 95% CI, 1.6% to 5.5%), colorectal cancer (2.0%; 95% CI, 0.7% to 3.4%), and breast cancer (1.2%; 95% CI, 0.1% to 2.3%) (Fig. 7). Point estimates and 95% CIs for all evaluated cancer sites are provided in Table S4.

When translated into estimated attributable case counts, the ranking differed somewhat from that based on PAF alone (Fig. 7 and Fig. S14). Lung cancer showed the largest estimated attributable burden (202 cases), followed by uterine cancer (183 cases), endometrial cancer (176 cases), and kidney cancer (160 cases). Estimated attributable case counts were also notable for colorectal cancer (118 cases), liver cancer (105 cases), and breast cancer (102 cases), despite their differing PAF values.

## Discussion

In this prospective cohort of more than 360,000 UK Biobank participants, CircS was associated with overall and site-specific incident cancer. Participants meeting the binary CircS definition had a 12% higher hazard of overall cancer, a modest individual-level association whose public-health relevance derives from the high prevalence of CircS. Findings were broadly consistent across Fine–Gray models, exclusion of early events, additional adjustment, and multiple imputation. Associations varied by cancer site, with the strongest positive estimates for endometrial, liver, and kidney cancers; the inverse estimates for prostate and brain cancer were exploratory.

The most substantial associations were observed for reproductive and digestive malignancies. Endometrial cancer, the most specific uterine outcome, showed the strongest association, and the broader uterine cancer category, which includes endometrial cancer, showed a consistent association; these nested outcomes reflect a single underlying association rather than 2 independent findings. Our mediation analysis provides a statistical rationale for this link, suggesting that SHBG may partly reflect the association between CircS and endometrial cancer. This finding is consistent with the established hypothesis that the metabolic components of CircS, such as visceral adiposity and hyperinsulinemia, suppress hepatic SHBG production, thereby increasing the bioavailability of unopposed circulating estrogens that drive endometrial cellular proliferation [33,34]. However, because SHBG was measured concurrently with the CircS components, this analysis cannot establish a causal mediating role. Furthermore, the nonlinear dose–response relationship for endometrial cancer indicates that risk escalates more steeply at higher levels of circadian-metabolic severity, highlighting the critical importance of managing severe syndrome clusters.

The statistical association of liver enzymes alanine aminotransferase and GGT with the CircS–liver cancer relationship, estimated in separate single-mediator models at 46.3% and 44.4%, respectively, suggests that CircS may serve as a marker of nonalcoholic fatty liver disease and systemic metabolic dysfunction, which represent a hepatometabolic pathway to carcinogenesis alongside other established etiologic routes, particularly chronic viral liver disease [35–37]. This systemic disruption extends to other digestive organs, with positive associations observed for esophageal, stomach, and colorectal cancers. Given the high prevalence of CircS in the study population, these findings suggest that a nontrivial proportion of digestive and reproductive cancers is statistically attributable to circadian-metabolic dysregulation under the model assumptions, and that integrated management of circadian-metabolic health merits evaluation as a potential prevention strategy.

Systemic inflammation appears to be a critical unifying mechanism linking circadian disruption to a broad spectrum of malignancies [38]. CRP and WBC count were statistically associated with overall cancer risk and specifically with lung cancer, which is consistent with prior evidence highlighting the predictive value of immune-inflammatory indices for cancer incidence [39]. While lung cancer exhibited a positive association overall, the risk appeared to plateau at higher levels of CircS severity and was notably stronger among ever-smokers. This suggests that the chronic, low-grade inflammation induced by CircS may synergize with exogenous carcinogens to promote a microenvironment conducive to tumor development [40,41]. The inclusion of depression as a CircS component is further supported by a recent individual participant data meta-analysis of 18 prospective cohorts, which found that depression and anxiety were associated with a modestly increased risk of overall and several site-specific cancers [42].

A distinctive feature of our findings was the inverse association observed for prostate cancer (HR, 0.94) and brain cancer (HR, 0.84). For prostate cancer, the lower observed incidence may reflect detection bias rather than a true protective effect: prostate-specific antigen testing patterns, obesity-related hemodilution of prostate-specific antigen, and differences in healthcare use can all influence diagnosis. One possible explanation is the epidemiological “metabolic paradox”, wherein the specific hormonal shifts associated with severe metabolic syndrome—particularly reduced circulating free testosterone levels secondary to obesity—may inadvertently suppress the initiation or growth of androgen-dependent prostate tumors [43]. Nevertheless, this epidemiological pattern should not be interpreted as evidence that circadian-metabolic dysfunction is uniformly protective because prostate cancer biology is shaped by complex interactions among androgen receptor signaling, metabolic and epigenetic plasticity, regulated cell death, and the tumor immune microenvironment [44–47]. For brain cancer, the inverse association was present in binary analyses but attenuated toward the null in continuous models; given the relatively limited number of events and this inconsistency across exposure metrics, the finding should be regarded as exploratory and does not support inference of a physiological threshold.

Our findings extend and are complemented by 2 recent studies. Pan et al. [22] reported that CircS predicted all-cause and cause-specific mortality, including cancer mortality, more strongly than the metabolic syndrome in both NHANES and the UK Biobank, consistent with the incremental prognostic value of the circadian components. Bai et al. [23] found that the association between CircS and cancer in middle-aged and elderly Chinese adults was partly mediated by the triglyceride-glucose index, a marker of insulin resistance. The mediation pattern we observed—dominated by sex-hormone-binding globulin for endometrial cancer and by inflammatory markers for overall and lung cancer—differs from the insulin-resistance-centered mechanism in that cohort, which may reflect differences in population ancestry, cancer spectrum, or the biomarkers available. Together, these studies extend the relevance of CircS from cancer incidence to mortality and from European to East Asian populations, and they underscore the need for cross-ancestry validation of both the association and its mediating pathways.

From a public health perspective, the PAF analysis indicates a nontrivial population burden statistically associated with CircS. Under the assumptions of the fitted competing-risk model, an estimated 13.5% of endometrial cancer cases and 13.4% of liver cancer cases in this cohort were attributable to CircS. Although the relative risk for more common cancers such as colorectal and breast cancer was more modest, their high baseline incidence resulted in substantial absolute attributable case counts. Because CircS is a composite state defined by 7 interrelated components rather than a single exposure that can be removed at once, these estimates should not be read as the number of cases that a single preventive action would avert. They nevertheless suggest that integrated interventions targeting both metabolic stability and circadian hygiene merit evaluation as a potential prevention strategy for cancer.

The strengths of this study include its large-scale prospective design, comprehensive evaluation of 23 cancer sites, and use of complementary Cox, competing-risk, and mediation frameworks. Several limitations should be acknowledged. First, CircS was assessed at baseline only and may not capture longitudinal changes; moreover, the binary definition used short sleep whereas the continuous severity score used bidirectional deviation from 7.5 h, so these exposure metrics should be interpreted as complementary rather than interchangeable. Second, sleep duration and depressive symptoms were partly self-reported, which may have introduced nondifferential misclassification. Third, residual confounding by unmeasured or imperfectly measured factors cannot be excluded, even after site-targeted adjustment. Fourth, the concurrent baseline measurement of CircS components and candidate biomarkers precludes establishing the temporal ordering required for causal mediation; the estimated proportions therefore represent a statistical decomposition of associations. Fifth, a substantial proportion of potentially eligible participants were excluded because of missing data. Although multiple imputation of missing covariates yielded materially consistent associations, selection bias related to data unavailable for exposure construction cannot be excluded. The UK Biobank healthy-volunteer selection, the predominantly White analytic cohort, and the restricted recruitment age range also limit generalizability. PAF estimates should therefore be interpreted as statistical attribution within a population resembling this cohort, not as an achievable preventive yield from a single intervention on the composite CircS state. Finally, despite Benjamini–Hochberg correction, the number of analyses means that borderline or exploratory findings may represent false positives [48].

In conclusion, CircS was associated with incident cancer risk, driven by a complex interplay of hormonal dysregulation, chronic inflammation, and metabolic impairment. These findings highlight the potential value of a holistic approach to cancer prevention research that integrates the maintenance of biological rhythms with metabolic health management.

## Methods

The UK Biobank study was approved by the North West Multi-centre Research Ethics Committee, and all participants provided written informed consent. UK Biobank data were accessed through application number 80787. This study followed the Strengthening the Reporting of Observational Studies in Epidemiology reporting guidelines.

### Study cohort

This study was conducted within the UK Biobank, a large population-based prospective cohort study that recruited approximately 500,000 adults across the UK between 2006 and 2010 [31,49]. At enrollment, participants completed touchscreen questionnaires and nurse-led interviews and underwent physical measurements and biological sample collection. For the present analysis, baseline assessment was defined as time zero for follow-up, and participants in the analytic sample were aged 37 to 73 years at baseline. Participants were excluded if they had prevalent cancer at or before baseline, lacked the information required to derive the CircS components, had missing key covariates required for the fully adjusted models, or had zero or negative follow-up time. Incident cancer outcomes were ascertained through linkage to national health records. Sex-specific cancers were evaluated in sex-restricted subcohorts.

### Exposure assessment

The primary exposure was CircS, defined as the presence of at least 4 of 7 component traits [6]. Secondary exposure measures included the CircS score (range, 0 to 7) and a standardized CircS severity *z* score reflecting the overall burden of circadian and metabolic dysregulation. For the continuous severity score, sleep was modeled as the absolute deviation from 7.5 h per night because both short and long sleep have been associated with adverse outcomes in a U-shaped manner [50]; the binary CircS definition used the established short-sleep threshold of less than 6 h. These measures therefore captured related but not identical aspects of sleep. All exposure metrics were evaluated in the same outcome-specific analytic populations.

CircS was ascertained from baseline anthropometric, biochemical, questionnaire, medication, and diagnostic-code data using a hierarchical approach. Central obesity was defined as a waist circumference of at least 102 cm in men or at least 88 cm in women. Elevated triglycerides were defined as triglycerides of at least 1.7 mmol/L, lipid-lowering medication use, or prior hyperlipidemia. Reduced HDL-C was defined as HDL-C below 1.0 mmol/L in men or below 1.3 mmol/L in women, or lipid-lowering treatment. Elevated blood pressure was defined as systolic blood pressure of at least 130 mm Hg, diastolic blood pressure of at least 85 mm Hg, antihypertensive treatment, or prior hypertension. Impaired glucose regulation was defined as fasting glucose of at least 5.6 mmol/L among participants fasting at least 8 h, glycated hemoglobin of at least 39 mmol/mol, antidiabetic treatment, or prior diabetes. Short sleep duration was defined as self-reported sleep duration of less than 6 h per day. Depression was defined at baseline by self-reported history, antidepressant use, mood disorder codes, or a Patient Health Questionnaire-9 score of 5 or higher. A CircS component was considered present if any qualifying source indicated a positive finding.

The continuous CircS severity *z* score was constructed by summing 7 individually standardized components—waist circumference, log-transformed triglycerides, inverted HDL-C, mean arterial pressure, glycated hemoglobin, sleep deviation from 7.5 h, and depression-related burden—and then re-standardizing the composite to a mean of 0 and an SD of 1. Associations for this exposure are reported per 1-SD increase.

### Outcome ascertainment

Participants were followed from baseline until the first occurrence of incident cancer, death, loss to follow-up, or administrative censoring on 2022 October 31, whichever came first. Incident cancers were identified from national cancer registry records using International Classification of Diseases, 10th Revision codes. The primary outcome was overall incident cancer, defined as malignant neoplasms (C00 to C97) excluding nonmelanoma skin cancer (C44). Secondary outcomes were 23 site-specific cancers, including head and neck, esophageal, stomach, colorectal, liver, pancreatic, lung, melanoma, soft tissue, breast, cervical, endometrial, uterine, ovarian, prostate, kidney, bladder, brain, thyroid, lymphoma, non-Hodgkin lymphoma, multiple myeloma, and leukemia. Detailed definitions of all exposures and outcomes, including the International Classification of Diseases, 10th Revision codes for each site-specific cancer, are provided in Table S5.

### Covariates

To reduce potential confounding, the adjusted models included baseline age, sex, ethnicity, smoking status, alcohol intake, Townsend deprivation index, and physical activity. In sex-restricted analyses, sex was omitted from the model. Detailed definitions of all covariates are provided in Table S6. In sensitivity analyses, BMI and CRP were additionally included.

### Statistical analysis

Baseline characteristics were summarized according to binary CircS status. Continuous variables are presented as mean (SD) or median (interquartile range), as appropriate, and categorical variables as number (percentage). Differences between groups were compared using the Wilcoxon rank-sum test for continuous variables and the Pearson *χ*^2^ test for categorical variables.

Associations between CircS and incident cancer were primarily evaluated using Cox proportional-hazards regression models, with results reported as HRs and 95% CIs. Participants were followed from baseline until the first occurrence of the outcome of interest, death, loss to follow-up, or administrative censoring, whichever came first. The primary exposure was binary CircS status, and the primary analysis focused on the fully adjusted model. Complementary analyses additionally modeled CircS as a continuous score and as a standardized severity *z* score. Separate Cox models were fitted for overall cancer and each site-specific cancer outcome. The proportional-hazards assumption was assessed using Schoenfeld-residual-based diagnostic tests.

Because death from causes other than cancer may preclude the occurrence of the cancer outcome of interest, competing-risk analyses were performed using Fine–Gray subdistribution hazard models, with noncancer death treated as the competing event [30]. Results are reported as sdHRs and 95% CIs. The covariate adjustment strategy paralleled that used in the Cox models. Benjamini–Hochberg false discovery rate correction was applied uniformly to all multiple-comparison families, with adjusted *P* values reported throughout [48]. Additional Fine–Gray and Cox models evaluated the 7 individual CircS components; each component was entered separately into an otherwise identically adjusted model.

Several sensitivity analyses were conducted in both the Cox and Fine–Gray frameworks. The primary fully adjusted models were repeated after multiple imputation of missing covariates, using the same exposure and covariate specifications. Site-targeted models additionally included smoking pack-years for lung cancer, menopausal status and hormone replacement therapy use for endometrial cancer, and chronic liver disease or viral hepatitis status plus alcohol intake frequency for liver cancer. To reduce the potential influence of reverse causation, analyses were repeated after excluding participants who developed cancer within the first 2 years of follow-up. A further-adjusted model additionally included BMI and CRP.

Potential dose–response associations between the CircS severity *z* score and cancer outcomes were examined using restricted cubic spline functions within Cox proportional-hazards models. Four knots were placed at the 5th, 35th, 65th, and 95th percentiles of the *z*-score distribution, with the median value used as the reference. Overall and nonlinear associations were evaluated on the basis of the spline terms.

Prespecified subgroup analyses were conducted to assess the consistency of the associations across clinically relevant strata, and heterogeneity was evaluated by testing multiplicative interaction terms. Mediation analyses were performed for overall cancer and selected site-specific cancers using candidate biomarkers identified in the mediation analysis pipeline. We used a counterfactual-based mediation framework to decompose the total association between CircS and incident cancer into direct and indirect components. Because CircS components and biomarkers were measured concurrently at baseline, the temporal ordering required for causal mediation cannot be established; the estimated proportions therefore represent a statistical decomposition of associations under the assumption of no unmeasured confounding, rather than causal mediating effects. Results are summarized primarily as the proportion of the total association statistically explained by each biomarker. PAFs were calculated from the adjusted sdHR from the fully adjusted Fine–Gray model and the observed prevalence of CircS (Pe) as PAF = Pe × (sdHR − 1) / [1 + Pe × (sdHR − 1)]; 95% CIs were obtained by applying the same transformation to the lower and upper 95% confidence limits of the sdHR, with Pe treated as fixed.

All analyses were performed using R software, version 4.4.1 (R Foundation for Statistical Computing). All statistical tests were 2-sided, and *P* <0.05 was considered statistically significant unless otherwise specified.

## Ethical Approval

This study was performed in accordance with the principles of the Declaration of Helsinki. The UK Biobank has full ethical approval from the NHS National Research Ethics Service (16/NW/0274), and informed consent was obtained before data collection from each participant. This research has been conducted using the UK Biobank Resource under Application Number 80787.

## Data Availability

Data from the UK Biobank (ukbiobank.ac.uk/) are available to all researchers on making an application.
