# Supplementary material for: Association of Circadian Syndrome with Incident Risk of 23 Site-Specific Cancers: A Large-Scale Prospective Study in the UK Biobank
Source: Research (Wash D C). 2026 Sep 22;9:1426. doi: 10.34133/research.1426 (PMC13594417; doi:10.34133/research.1426)
Supplement: Supplementary file 1 [file research.1426.f1.zip › Supplemental Figures.pdf]

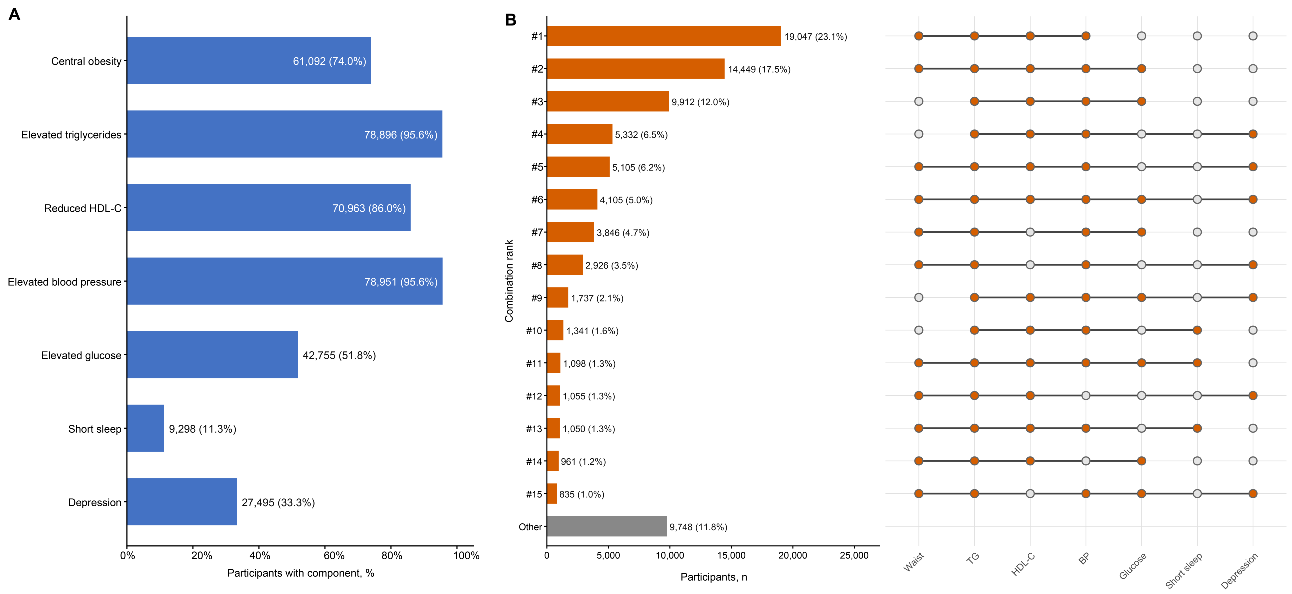

Figure S1. Distribution of circadian syndrome components and the most common exact component combinations among participants with circadian syndrome. (A) Prevalence of each of the seven components among 82,547 participants meeting the binary CircS definition. (B) Counts and proportions for the 15 most common exact component combinations; filled orange circles indicate components present and open circles indicate components absent. The 15 combinations accounted for 88.2% of participants with CircS. BP, blood pressure; CircS, circadian syndrome; HDL-C, high-density lipoprotein cholesterol; TG, triglycerides.

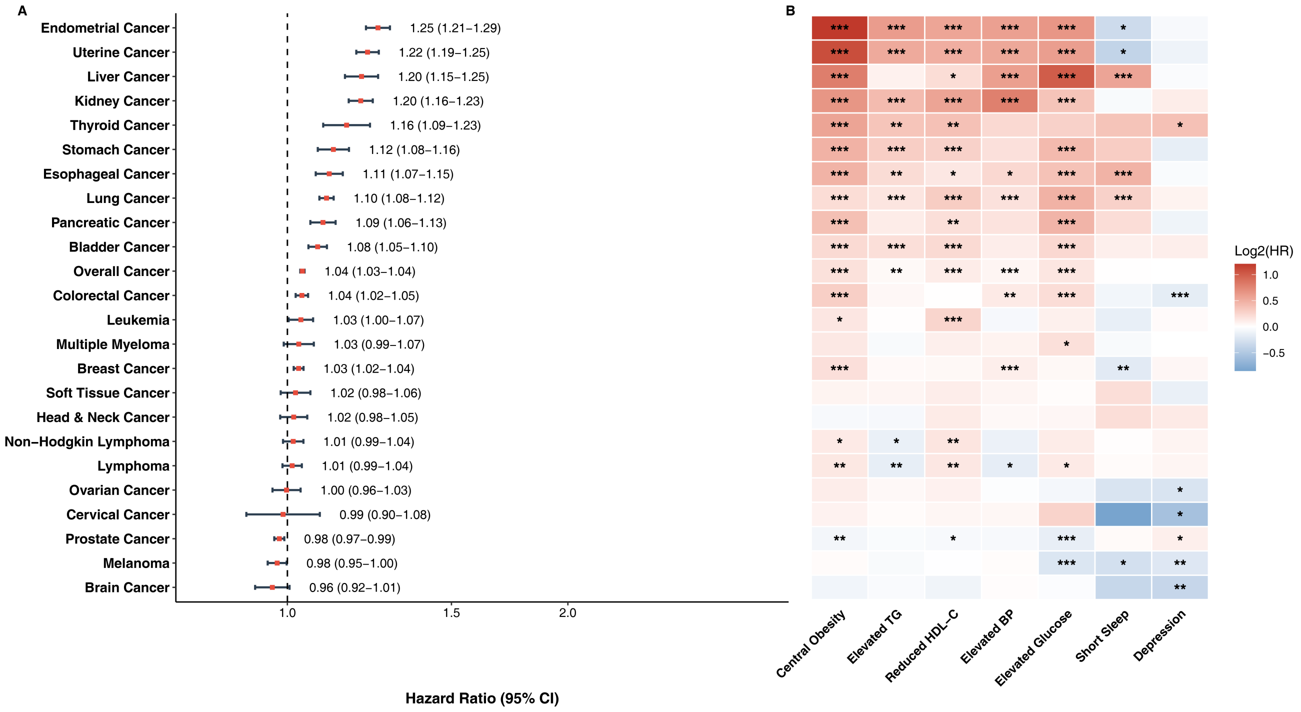

Figure S2. Associations of Circadian Syndrome Score and Individual CircS Components With Overall and Site-Specific Incident Cancer Risk. (A) shows HRs and 95% CIs for the associations of the CircS score (range, 0-7) with overall and site-specific incident cancers in fully adjusted Cox proportional hazards models. Estimates are presented per 1-point increment in CircS score. The dashed vertical line indicates the null value (HR = 1.0). (B) shows the corresponding associations of individual CircS components with overall and site-specific cancer outcomes in fully adjusted Cox models, displayed as log2(HR). Warmer colors indicate positive associations and cooler colors indicate inverse associations. CircS components included central obesity, elevated TG, reduced HDL-C, elevated BP, elevated glucose, short sleep, and depression.

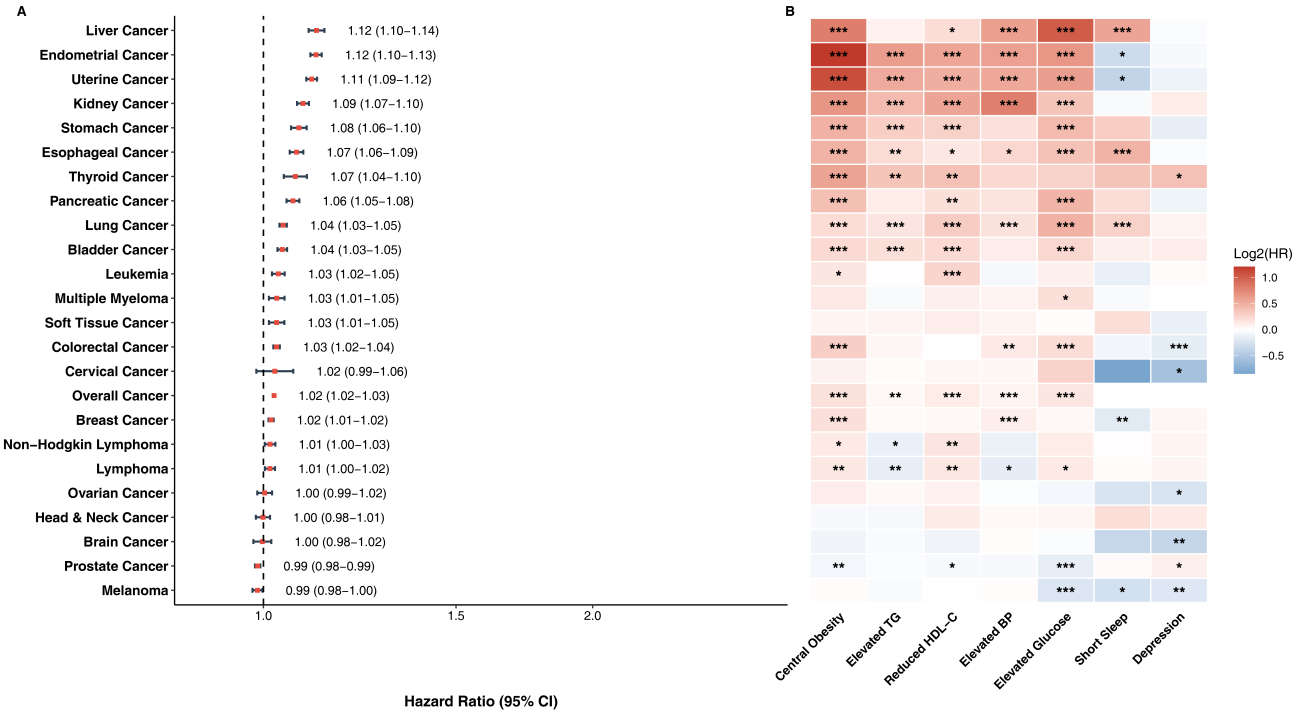

Figure S3. Associations of Circadian Syndrome Severity z Score and Individual CircS Components With Overall and Site-Specific Incident Cancer Risk. (A) shows HRs and 95% CIs for the associations of the standardized CircS severity z score with overall and site-specific incident cancers in fully adjusted Cox proportional hazards models. Estimates are presented per 1-SD increase in the CircS severity z score. The dashed vertical line indicates the null value (HR = 1.0). (B) shows the corresponding associations of individual CircS components with overall and site-specific cancer outcomes in fully adjusted Cox models, displayed as log2(HR). Warmer colors indicate positive associations and cooler colors indicate inverse associations. CircS components included central obesity, elevated TG, reduced HDL-C, elevated BP, elevated glucose, short sleep, and depression.

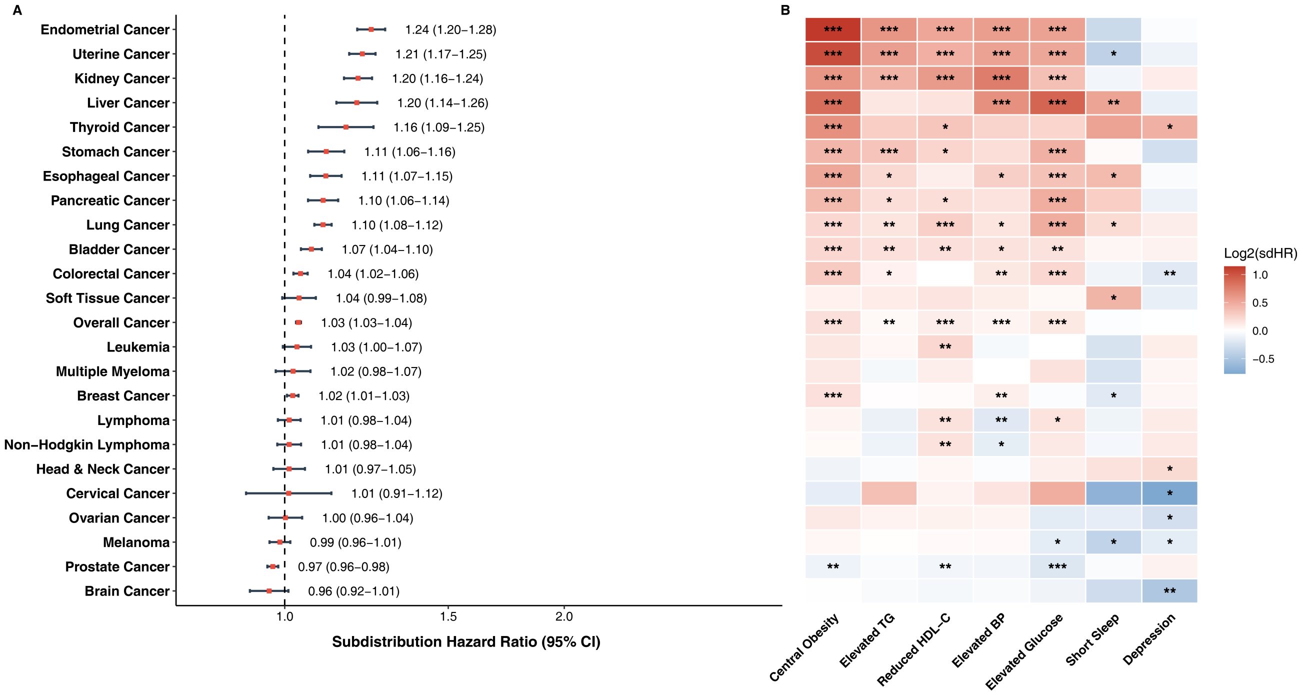

Figure S4. Associations of circadian syndrome score and individual CircS components with overall and site-specific incident cancer in Fine-Gray models. (A) shows sdHRs and 95% CIs for the associations of the CircS score (range, 0-7) with overall and site-specific incident cancers in fully adjusted Fine-Gray models, with noncancer death treated as the competing event. Estimates are presented per 1-point increment in CircS score. The dashed vertical line indicates the null value (sdHR = 1.0). (B) shows the corresponding associations of individual CircS components with overall and site-specific cancer outcomes in fully adjusted Fine-Gray models, displayed as log2(sdHR). Warmer colors indicate positive associations and cooler colors indicate inverse associations. CircS components included central obesity, elevated TG, reduced HDL-C, elevated BP, elevated glucose, short sleep, and depression.

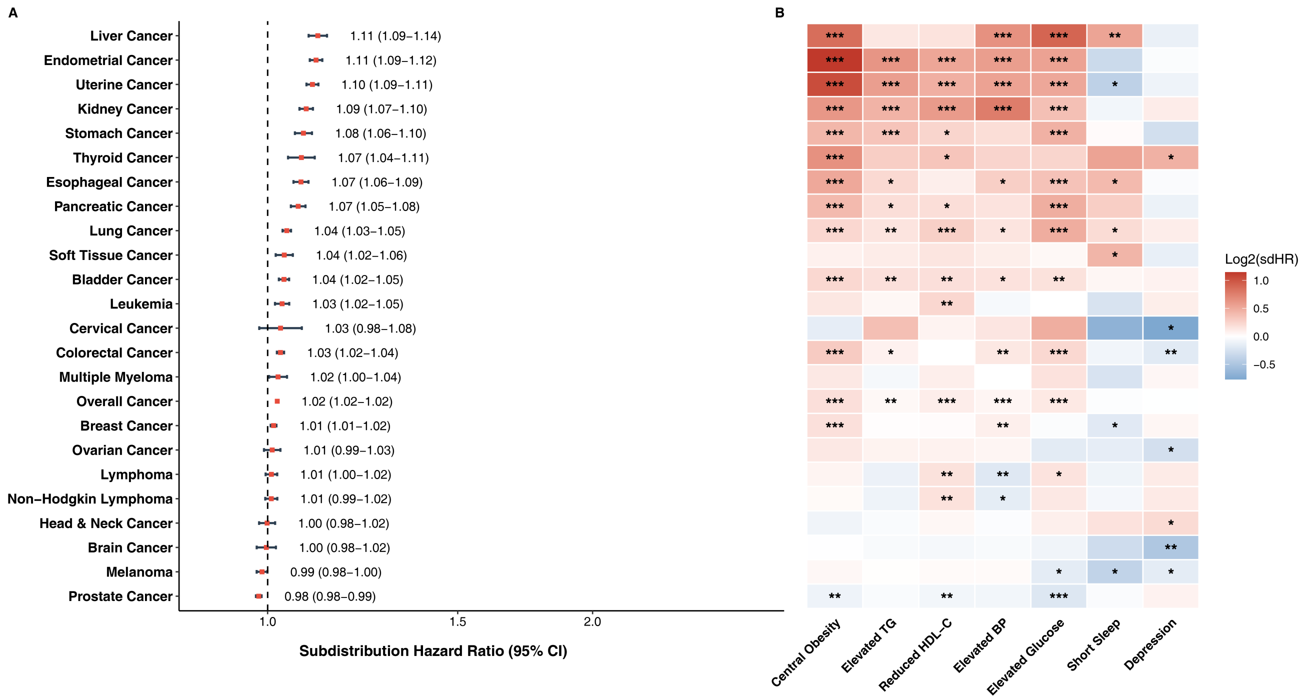

Figure S5. Associations of circadian syndrome severity z score and individual CircS components with overall and site-specific incident cancer in Fine-Gray models. (A) shows sdHRs and 95% CIs for the associations of the standardized CircS severity z score with overall and site-specific incident cancers in fully adjusted Fine-Gray models, with noncancer death treated as the competing event. Estimates are presented per 1-SD increase in the CircS severity z score. The dashed vertical line indicates the null value (sdHR = 1.0). (B) shows the corresponding associations of individual CircS components with overall and site-specific cancer outcomes in fully adjusted Fine-Gray models, displayed as log2(sdHR). Warmer colors indicate positive associations and cooler colors indicate inverse associations. CircS components included central obesity, elevated TG, reduced HDL-C, elevated BP, elevated glucose, short sleep, and depression.

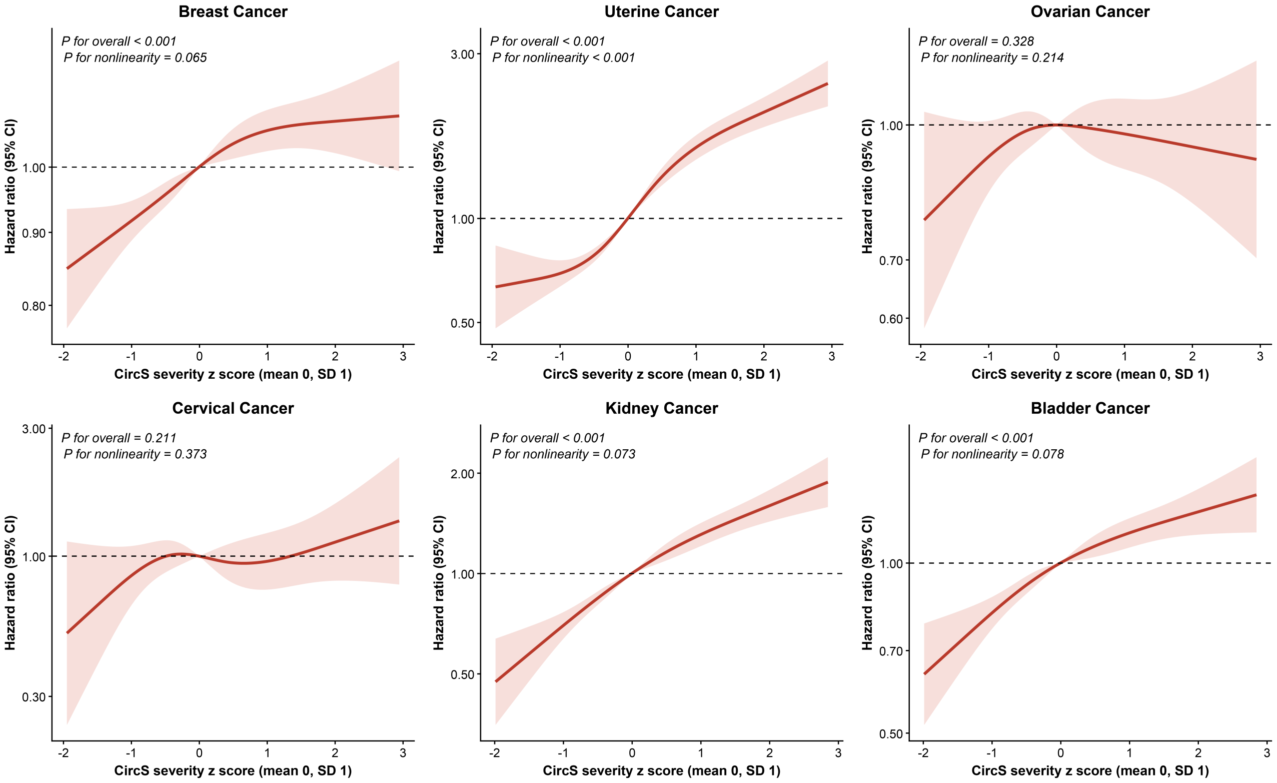

Figure S6. Restricted cubic spline analyses of circadian syndrome severity and additional site-specific cancers. Restricted cubic spline curves showing the associations between the standardized CircS severity z score and additional site-specific incident cancers in fully adjusted Cox proportional hazards models. This figure includes breast, uterine, ovarian, cervical, kidney, and bladder cancers. Knots were placed at the 5th, 35th, 65th, and 95th percentiles of the z-score distribution, with the median z score used as the reference. Solid lines indicate HRs, and shaded areas indicate 95% CIs. P values for the overall association and for nonlinearity are shown in each panel.

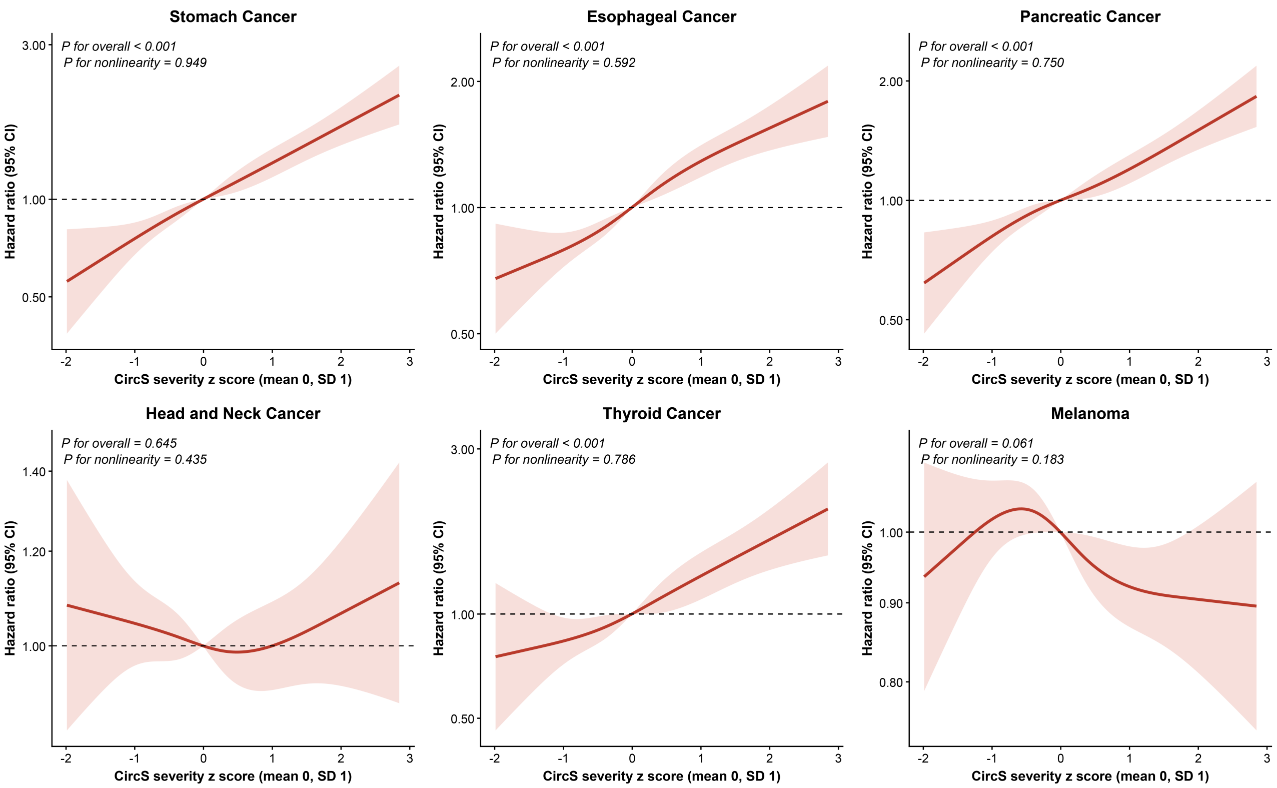

Figure S7. Restricted cubic spline analyses of circadian syndrome severity and additional site-specific cancers. Restricted cubic spline curves showing the associations between the standardized CircS severity z score and additional site-specific incident cancers in fully adjusted Cox proportional hazards models. This figure includes stomach, esophageal, pancreatic, head and neck, thyroid, and melanoma outcomes. Knots were placed at the 5th, 35th, 65th, and 95th percentiles of the z-score distribution, with the median z score used as the reference. Solid lines indicate HRs, and shaded areas indicate 95% CIs. P values for the overall association and for nonlinearity are shown in each panel.

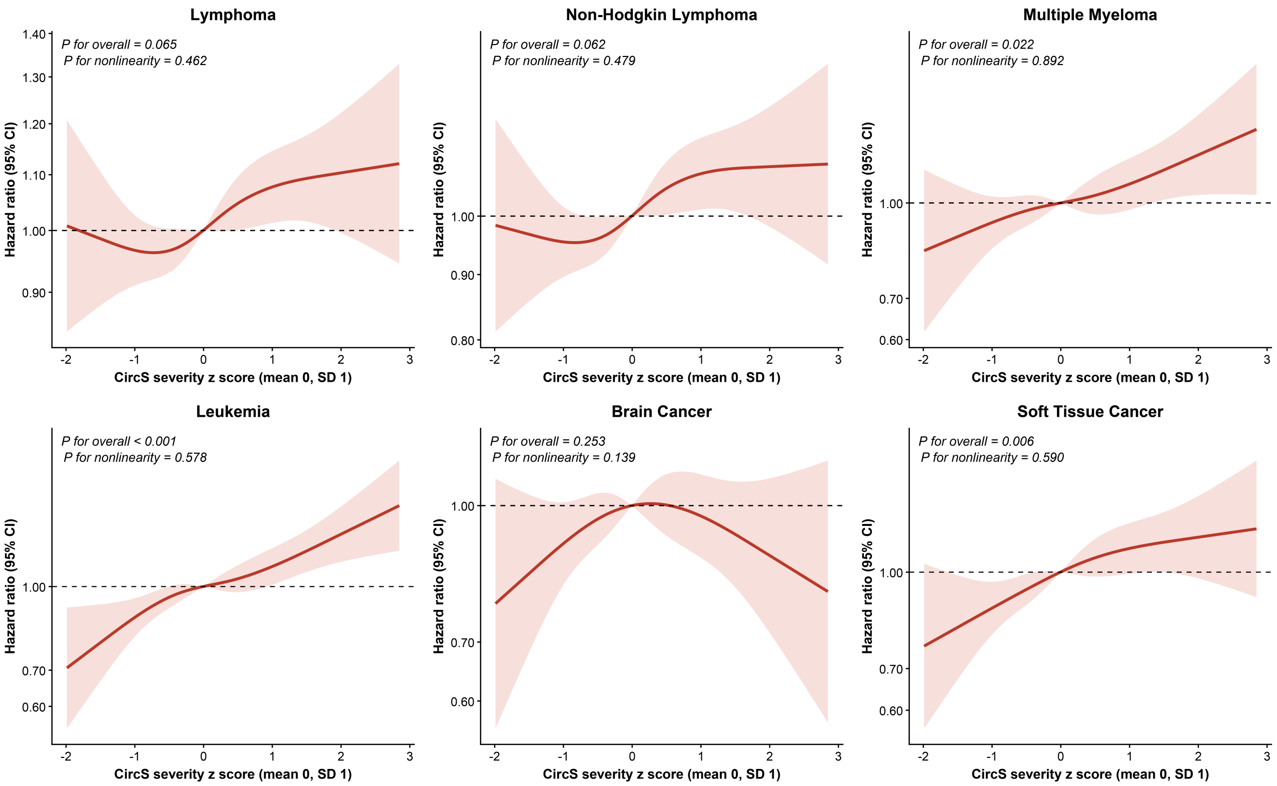

Figure S8. Restricted cubic spline analyses of circadian syndrome severity and additional site-specific cancers. Restricted cubic spline curves showing the associations between the standardized CircS severity z score and additional site-specific incident cancers in fully adjusted Cox proportional hazards models. This figure includes lymphoma, non-Hodgkin lymphoma, multiple myeloma, leukemia, brain cancer, and soft tissue cancer. Knots were placed at the 5th, 35th, 65th, and 95th percentiles of the z-score distribution, with the median z score used as the reference. Solid lines indicate HRs, and shaded areas indicate 95% CIs. P values for the overall association and for nonlinearity are shown in each panel.

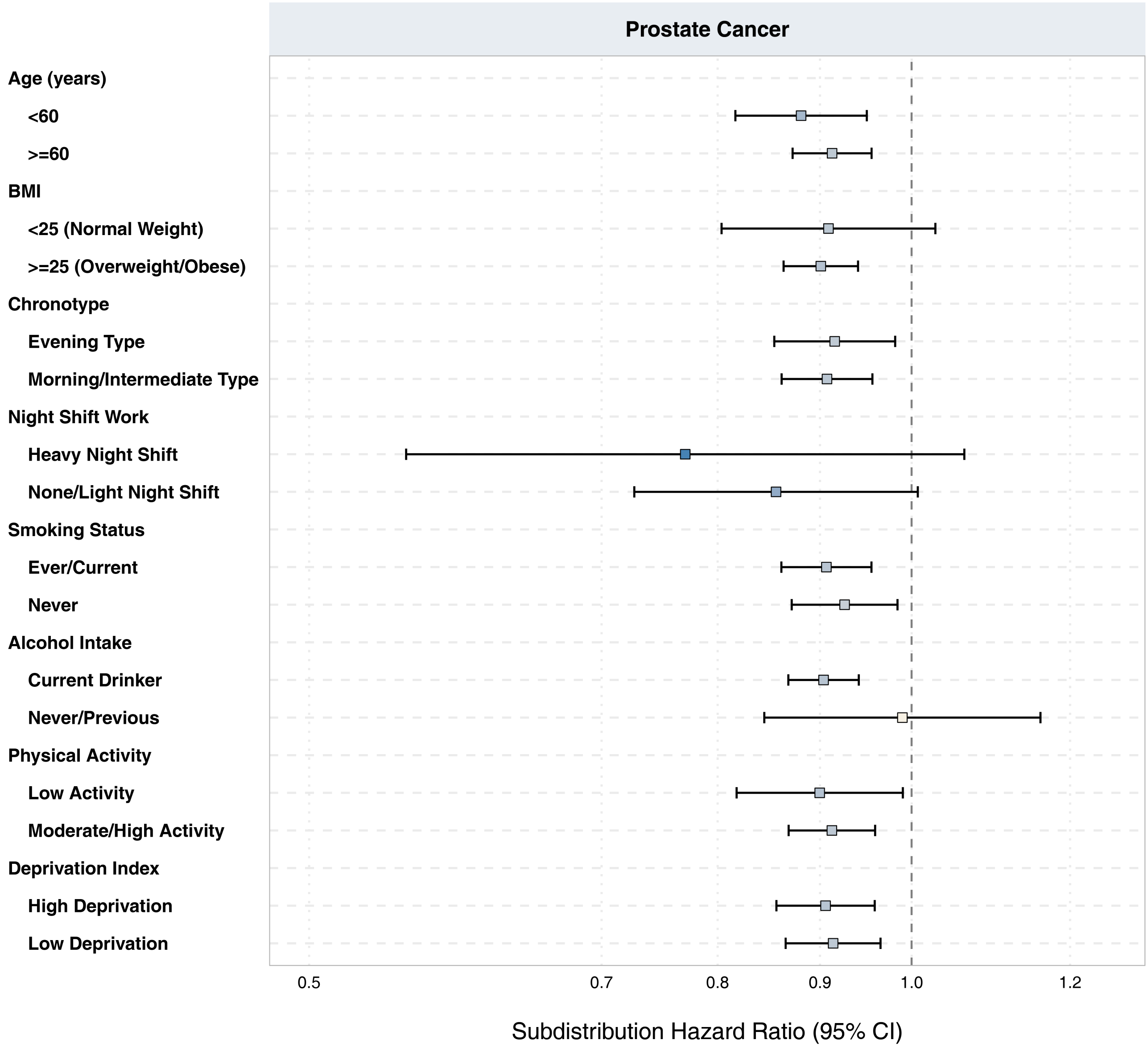

Figure S9. Subgroup associations of circadian syndrome with incident prostate cancer in Fine-Gray models. Forest plot showing subdistribution hazard ratios (sdHRs) and 95% CIs for the association of binary circadian syndrome (CircS) status with incident prostate cancer across prespecified subgroups in fully adjusted Fine-Gray models.

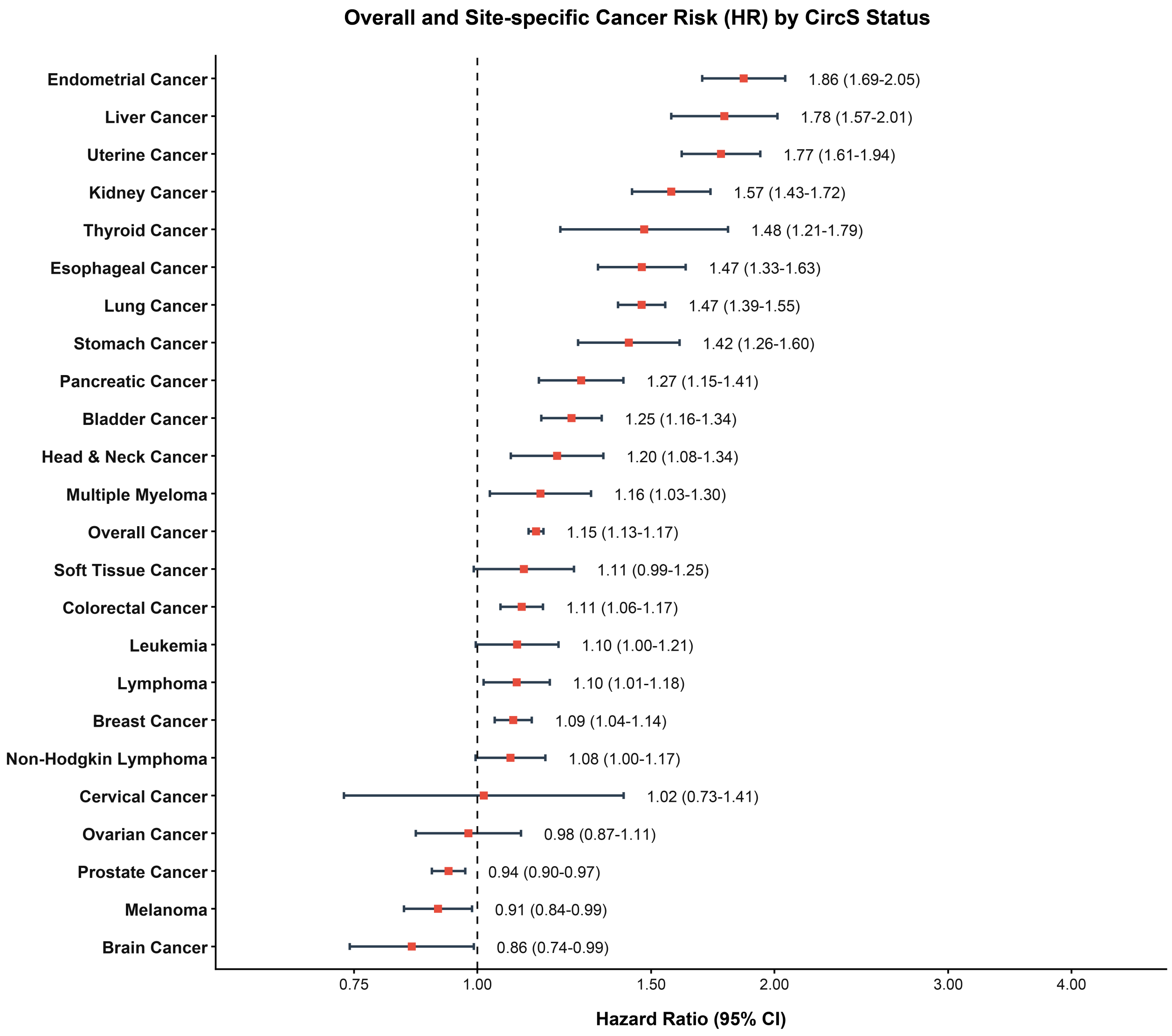

Figure S10. Multiple-imputation sensitivity analysis of the association between circadian syndrome and incident cancer in Cox models. Forest plot showing hazard ratios (HRs) and 95% CIs for the association of binary circadian syndrome (CircS) status (score ≥4 vs 0-3) with overall and site-specific incident cancers after multiple imputation of missing covariates. Models used the same covariate adjustment set as the primary Cox analyses. The dashed vertical line indicates the null value (HR = 1.0).

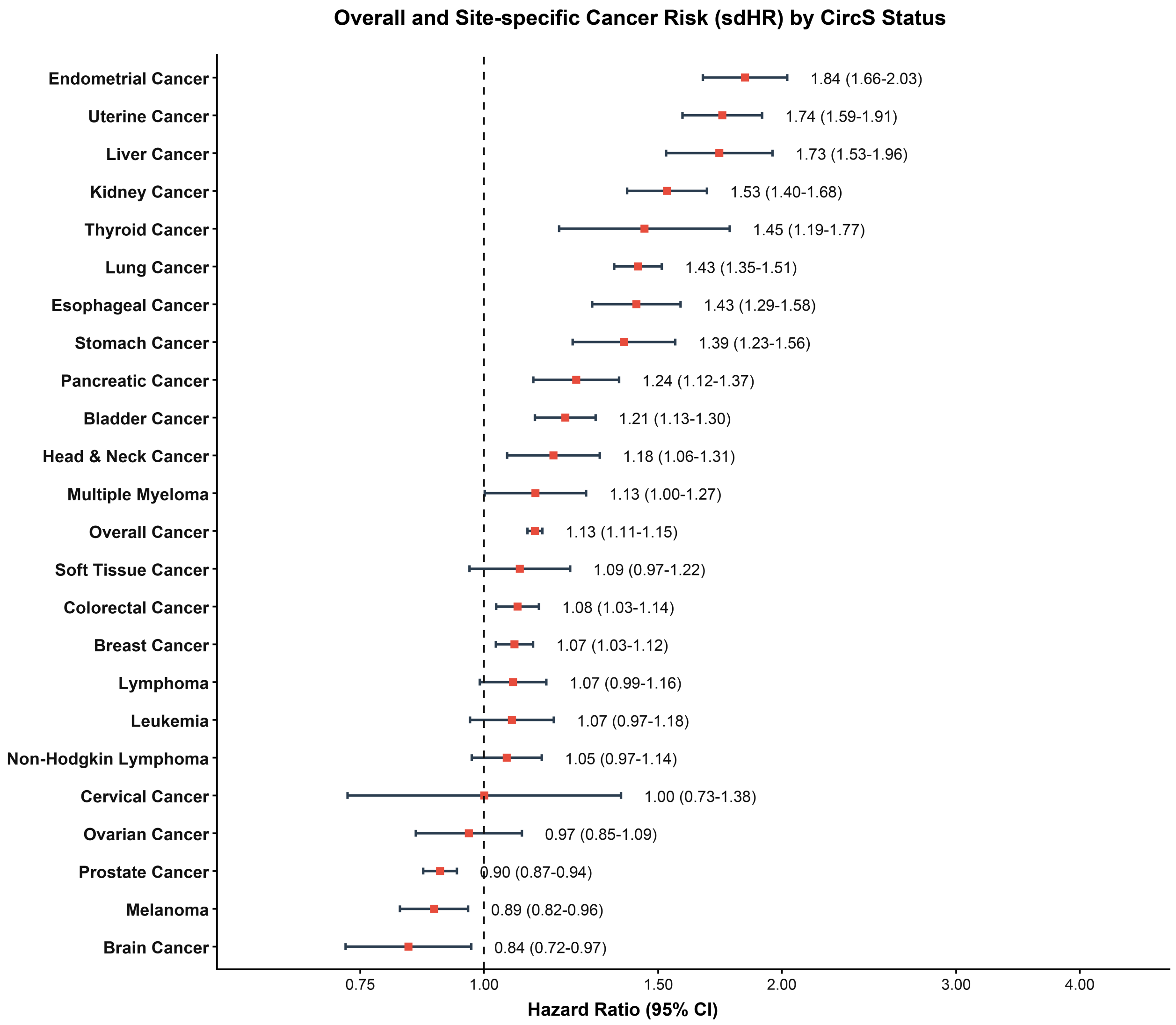

Figure S11. Multiple-imputation sensitivity analysis of the association between circadian syndrome and incident cancer in Fine-Gray models. Forest plot showing subdistribution hazard ratios (sdHRs) and 95% CIs for the association of binary circadian syndrome (CircS) status (score ≥4 vs 0-3) with overall and site-specific incident cancers after multiple imputation of missing covariates, with noncancer death treated as the competing event. Models used the same covariate adjustment set as the primary Fine-Gray analyses. The dashed vertical line indicates the null value (sdHR = 1.0).

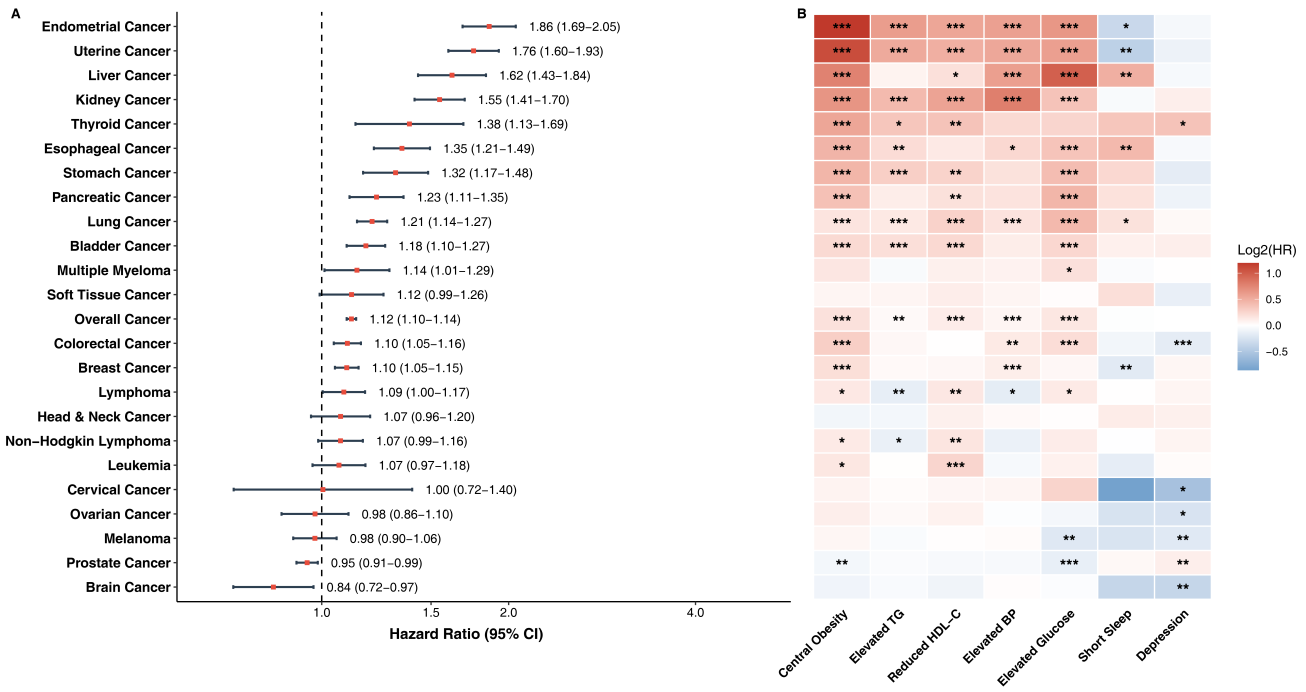

Figure S12. Sensitivity analysis excluding incident cancers diagnosed within the first 2 years of follow-up. (A) shows hazard ratios (HRs) and 95% CIs for the associations of binary circadian syndrome (CircS) status with overall and site-specific incident cancers in fully adjusted Cox proportional hazards models after excluding participants who developed cancer within the first 2 years of follow-up. (B) shows the corresponding associations of individual CircS components with overall and site-specific cancer outcomes, displayed as log2(HR). Warmer colors indicate positive associations and cooler colors indicate inverse associations. CircS components included central obesity, elevated triglycerides, reduced high-density lipoprotein cholesterol, elevated blood pressure, elevated glucose, short sleep, and depression.

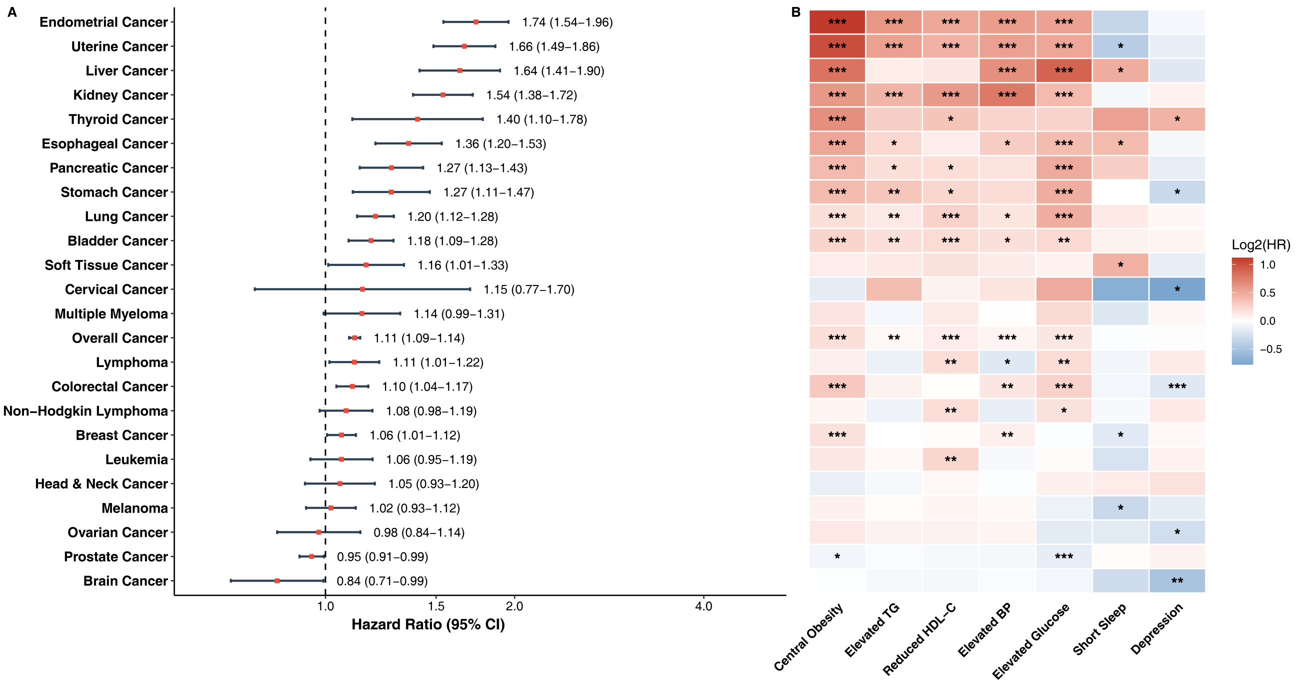

Figure S13. Sensitivity analysis with additional adjustment for body mass index and C-reactive protein. (A) shows hazard ratios (HRs) and 95% CIs for the associations of binary circadian syndrome (CircS) status with overall and site-specific incident cancers in Cox proportional hazards models additionally adjusted for body mass index (BMI) and C-reactive protein (CRP). (B) shows the corresponding associations of individual CircS components with overall and site-specific cancer outcomes, displayed as log2(HR). Warmer colors indicate positive associations and cooler colors indicate inverse associations. CircS components included central obesity, elevated triglycerides, reduced high-density lipoprotein cholesterol, elevated blood pressure, elevated glucose, short sleep, and depression.

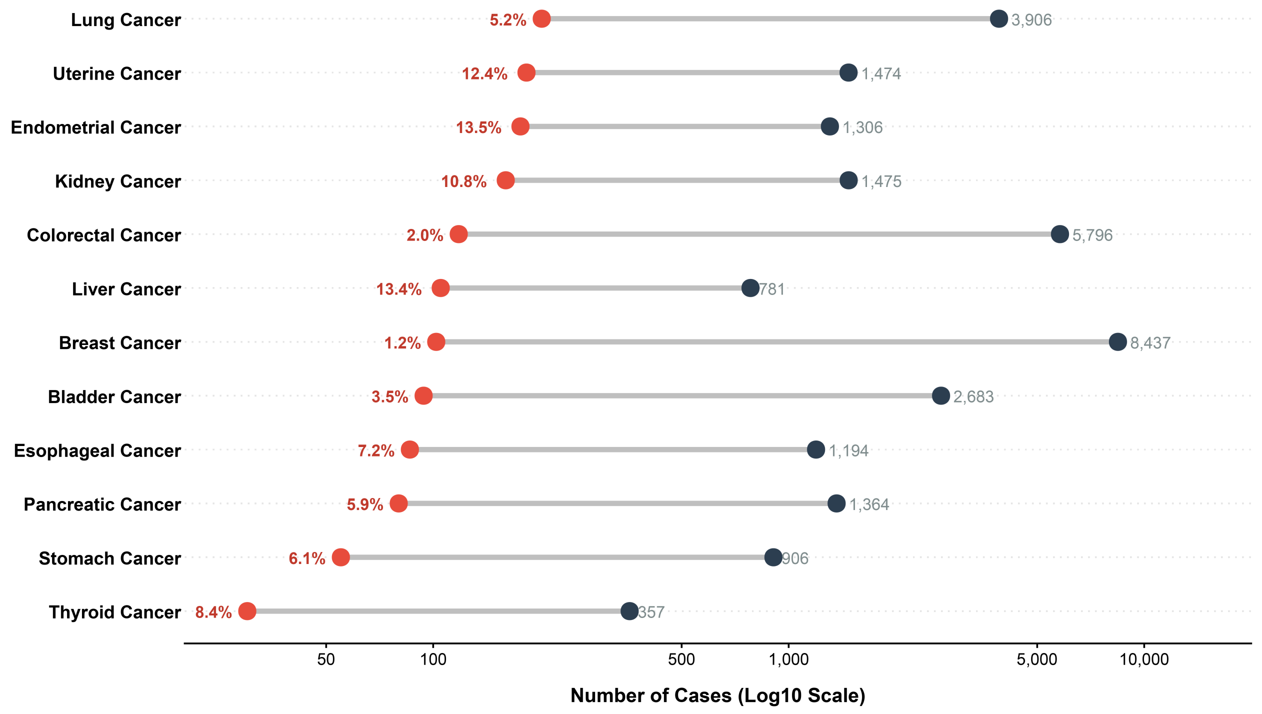

Figure S14. Total observed and estimated attributable case counts for selected cancers associated with circadian syndrome. Dumbbell plot comparing the total observed number of incident cases and the estimated attributable case counts for selected site-specific cancers associated with circadian syndrome (CircS). Dark points indicate total observed cases, red points indicate estimated attributable cases, and connecting lines represent the gap between overall event burden and estimated attributable burden. The x-axis is shown on a logarithmic scale. Corresponding PAF estimates are labeled for each cancer site.
