# Supplementary material for: Association of Circadian Syndrome with Incident Risk of 23 Site-Specific Cancers: A Large-Scale Prospective Study in the UK Biobank
Source: Research (Wash D C). 2026 Sep 22;9:1426. doi: 10.34133/research.1426 (PMC13594417; doi:10.34133/research.1426)
Supplement: Supplementary file 1 [file research.1426.f1.zip › Supplement_Tables.docx]

**Table S1. Numbers at risk, incident cases, person-years of follow-up, and crude incidence rates for overall and site-specific cancers, overall and according to circadian syndrome status**

***Panel A. Overall analytic population***

| **Cancer site** | **Analytic population** | **Participants at risk, No.** | **Incident cases, No.** | **Person-years of follow-up** | **Crude incidence rate*** |
| --- | --- | --- | --- | --- | --- |
| Overall cancer | Full cohort | 367,832 | 47,695 | 4,683,050.6 | 1,018.5 |
| Head and neck cancer | Full cohort | 367,832 | 1,233 | 4,900,214.6 | 25.2 |
| Esophageal cancer | Full cohort | 367,832 | 1,194 | 4,903,046.8 | 24.4 |
| Stomach cancer | Full cohort | 367,832 | 906 | 4,903,900.8 | 18.5 |
| Colorectal cancer | Full cohort | 367,832 | 5,796 | 4,879,748.7 | 118.8 |
| Liver cancer | Full cohort | 367,832 | 781 | 4,904,765.6 | 15.9 |
| Pancreatic cancer | Full cohort | 367,832 | 1,364 | 4,904,484.8 | 27.8 |
| Lung cancer | Full cohort | 367,832 | 3,906 | 4,898,125.7 | 79.7 |
| Melanoma | Full cohort | 367,832 | 2,653 | 4,891,586.4 | 54.2 |
| Soft tissue cancer | Full cohort | 367,832 | 1,007 | 4,903,274.2 | 20.5 |
| Breast cancer | Female cohort | 190,777 | 8,437 | 2,513,452.8 | 335.7 |
| Uterine cancer | Female cohort | 190,777 | 1,474 | 2,558,744.7 | 57.6 |
| Ovarian cancer | Female cohort | 190,777 | 1,006 | 2,562,998.1 | 39.3 |
| Prostate cancer | Male cohort | 177,055 | 10,186 | 2,286,358.5 | 445.5 |
| Kidney cancer | Full cohort | 367,832 | 1,475 | 4,899,852.5 | 30.1 |
| Bladder cancer | Full cohort | 367,832 | 2,683 | 4,892,752.0 | 54.8 |
| Brain cancer | Full cohort | 367,832 | 816 | 4,904,929.9 | 16.6 |
| Thyroid cancer | Full cohort | 367,832 | 357 | 4,903,900.0 | 7.3 |
| Lymphoma | Full cohort | 367,832 | 2,398 | 4,894,623.4 | 49.0 |
| Non-Hodgkin lymphoma | Full cohort | 367,832 | 2,160 | 4,895,745.3 | 44.1 |
| Multiple myeloma | Full cohort | 367,832 | 991 | 4,902,099.4 | 20.2 |
| Leukemia | Full cohort | 367,832 | 1,509 | 4,900,219.4 | 30.8 |
| Cervical cancer | Female cohort | 190,777 | 150 | 2,566,128.8 | 5.8 |
| Endometrial cancer | Female cohort | 190,777 | 1,306 | 2,559,468.6 | 51.0 |

***Panel B. According to circadian syndrome status***

| **Cancer site** | **CircS** | | | **No CircS** | | |
| --- | --- | --- | --- | --- | --- | --- |
|  | **Incident cases, No.** | **Person-years of follow-up** | **Crude incidence rate*** | **Incident cases, No.** | **Person-years of follow-up** | **Crude incidence rate*** |
| Overall cancer | 13,457 | 1,021,312.9 | 1,317.6 | 34,238 | 3,661,737.7 | 935.0 |
| Head and neck cancer | 347 | 1,079,508.3 | 32.1 | 886 | 3,820,706.3 | 23.2 |
| Esophageal cancer | 427 | 1,080,123.6 | 39.5 | 767 | 3,822,923.2 | 20.1 |
| Stomach cancer | 311 | 1,080,401.4 | 28.8 | 595 | 3,823,499.4 | 15.6 |
| Colorectal cancer | 1,653 | 1,073,611.3 | 154.0 | 4,143 | 3,806,137.4 | 108.9 |
| Liver cancer | 315 | 1,080,655.2 | 29.1 | 466 | 3,824,110.4 | 12.2 |
| Pancreatic cancer | 450 | 1,080,679.3 | 41.6 | 914 | 3,823,805.5 | 23.9 |
| Lung cancer | 1,383 | 1,078,503.0 | 128.2 | 2,523 | 3,819,622.7 | 66.1 |
| Melanoma | 644 | 1,077,648.8 | 59.8 | 2,009 | 3,813,937.6 | 52.7 |
| Soft tissue cancer | 299 | 1,080,380.4 | 27.7 | 708 | 3,822,893.8 | 18.5 |
| Breast cancer | 1,853 | 497,920.5 | 372.1 | 6,584 | 2,015,532.3 | 326.7 |
| Uterine cancer | 473 | 506,843.2 | 93.3 | 1,001 | 2,051,901.5 | 48.8 |
| Ovarian cancer | 225 | 508,598.5 | 44.2 | 781 | 2,054,399.6 | 38.0 |
| Prostate cancer | 2,791 | 557,561.0 | 500.6 | 7,395 | 1,728,797.5 | 427.8 |
| Kidney cancer | 537 | 1,078,887.6 | 49.8 | 938 | 3,820,964.9 | 24.5 |
| Bladder cancer | 893 | 1,076,663.7 | 82.9 | 1,790 | 3,816,088.3 | 46.9 |
| Brain cancer | 180 | 1,080,961.2 | 16.7 | 636 | 3,823,968.7 | 16.6 |
| Thyroid cancer | 101 | 1,080,638.1 | 9.3 | 256 | 3,823,261.9 | 6.7 |
| Lymphoma | 675 | 1,078,150.6 | 62.6 | 1,723 | 3,816,472.8 | 45.1 |
| Non-Hodgkin lymphoma | 598 | 1,078,475.1 | 55.4 | 1,562 | 3,817,270.2 | 40.9 |
| Multiple myeloma | 292 | 1,080,049.8 | 27.0 | 699 | 3,822,049.6 | 18.3 |
| Leukemia | 438 | 1,079,578.4 | 40.6 | 1,071 | 3,820,641.0 | 28.0 |
| Cervical cancer | 34 | 509,286.0 | 6.7 | 116 | 2,056,842.8 | 5.6 |
| Endometrial cancer | 434 | 506,977.8 | 85.6 | 872 | 2,052,490.8 | 42.5 |

***Note.*** Crude incidence rates are expressed per 100,000 person-years. For each outcome, person-years accrued from baseline until the first occurrence of the relevant cancer, death, loss to follow-up, or administrative censoring on October 31, 2022, whichever came first. Sex-specific cancers were evaluated in sex-restricted analytic populations (female: breast, uterine, ovarian, cervical, and endometrial cancers; male: prostate cancer). Uterine cancer includes endometrial cancer, and lymphoma includes non-Hodgkin lymphoma; these nested outcomes were analyzed separately and should not be interpreted as independent events. CircS, circadian syndrome.

**Table S2. Associations of circadian syndrome with selected site-specific incident cancers after site-targeted covariate adjustment**

| **Cancer site** | **Additional site-targeted covariates** | **Cox HR (95% CI)** | **P value** | **Fine-Gray sdHR (95% CI)** | **P value** |
| --- | --- | --- | --- | --- | --- |
| Lung cancer | Smoking pack-years | 1.20 (1.14–1.27) | <0.001 | 1.17 (1.11–1.24) | <0.001 |
| Endometrial cancer | Menopausal status and hormone replacement therapy use | 1.86 (1.69–2.06) | <0.001 | 1.84 (1.67–2.04) | <0.001 |
| Liver cancer | Chronic liver disease or viral hepatitis status and alcohol intake frequency | 1.63 (1.44–1.85) | <0.001 | 1.60 (1.41–1.81) | <0.001 |

Note. Values are hazard ratios (HRs) or subdistribution hazard ratios (sdHRs) with 95% confidence intervals for binary circadian syndrome (CircS; score ≥4 vs 0-3). Cox and Fine-Gray models retained the primary covariate set and additionally included the site-specific covariates shown; Fine-Gray models treated noncancer death as the competing event. CI, confidence interval; CircS, circadian syndrome; HR, hazard ratio; sdHR, subdistribution hazard ratio.

**Table S3. Mediation Analysis Results for the Associations Between Circadian Syndrome and Selected Incident Cancers**

| **Outcome** | **Mediator** | **No. included** | **Total HR** | **Direct HR** | **P value for mediation** | **Proportion mediated, %** |
| --- | --- | --- | --- | --- | --- | --- |
| ***Overall cancer*** | | | | | | |
|  | C-reactive protein | 343,082 | 1.112 | 1.081 | <0.001 | 26.4 |
|  | White blood cell count | 350,556 | 1.118 | 1.088 | <0.001 | 24.6 |
|  | Alanine aminotransferase | 343,690 | 1.113 | 1.128 | <0.001 | 0.0 |
|  | Gamma-glutamyl transferase | 343,817 | 1.113 | 1.098 | <0.001 | 12.4 |
|  | Insulin-like growth factor 1 | 342,156 | 1.112 | 1.115 | <0.001 | 0.0 |
|  | Sex hormone-binding globulin | 311,634 | 1.110 | 1.106 | 0.202 | 3.9 |
|  | Testosterone | 313,755 | 1.115 | 1.122 | <0.001 | 0.0 |
|  | Estradiol | 58,581 | 1.132 | 1.131 | 0.827 | 0.2 |
| ***Endometrial cancer*** | | | | | | |
|  | C-reactive protein | 177,385 | 1.795 | 1.556 | <0.001 | 24.4 |
|  | White blood cell count | 181,062 | 1.712 | 1.631 | <0.001 | 9.0 |
|  | Alanine aminotransferase | 177,609 | 1.786 | 1.715 | 0.0105 | 7.0 |
|  | Gamma-glutamyl transferase | 177,430 | 1.780 | 1.678 | <0.001 | 10.2 |
|  | Insulin-like growth factor 1 | 176,810 | 1.759 | 1.752 | 0.447 | 0.7 |
|  | Sex hormone-binding globulin | 159,132 | 1.753 | 1.317 | <0.001 | 51.0 |
|  | Testosterone | 149,110 | 1.836 | 1.824 | <0.001 | 1.1 |
|  | Estradiol | 44,430 | 2.138 | 2.144 | 0.797 | 0.0 |
| ***Liver cancer*** | | | | | | |
|  | C-reactive protein | 343,082 | 1.662 | 1.557 | <0.001 | 12.8 |
|  | White blood cell count | 350,556 | 1.653 | 1.631 | 0.285 | 2.7 |
|  | Alanine aminotransferase | 343,690 | 1.692 | 1.326 | <0.001 | 46.3 |
|  | Gamma-glutamyl transferase | 343,817 | 1.688 | 1.338 | <0.001 | 44.4 |
|  | Insulin-like growth factor 1 | 342,156 | 1.601 | 1.503 | <0.001 | 13.4 |
|  | Sex hormone-binding globulin | 311,634 | 1.700 | 2.155 | <0.001 | 0.0 |
|  | Testosterone | 313,755 | 1.711 | 1.797 | <0.001 | 0.0 |
|  | Estradiol | 58,581 | 1.423 | 1.430 | 0.288 | 0.0 |
| ***Kidney cancer*** | | | | | | |
|  | C-reactive protein | 343,082 | 1.551 | 1.445 | <0.001 | 16.0 |
|  | White blood cell count | 350,556 | 1.551 | 1.456 | <0.001 | 14.4 |
|  | Alanine aminotransferase | 343,690 | 1.572 | 1.539 | 0.131 | 4.6 |
|  | Gamma-glutamyl transferase | 343,817 | 1.558 | 1.509 | 0.00525 | 7.1 |
|  | Insulin-like growth factor 1 | 342,156 | 1.550 | 1.553 | 0.517 | 0.0 |
|  | Sex hormone-binding globulin | 311,634 | 1.566 | 1.459 | <0.001 | 15.9 |
|  | Testosterone | 313,755 | 1.584 | 1.571 | 0.355 | 1.8 |
|  | Estradiol | 58,581 | 1.601 | 1.599 | 0.779 | 0.4 |
| ***Lung cancer*** | | | | | | |
|  | C-reactive protein | 343,082 | 1.209 | 1.117 | <0.001 | 41.5 |
|  | White blood cell count | 350,556 | 1.207 | 1.105 | <0.001 | 47.0 |
|  | Alanine aminotransferase | 343,690 | 1.206 | 1.250 | <0.001 | 0.0 |
|  | Gamma-glutamyl transferase | 343,817 | 1.214 | 1.178 | <0.001 | 15.5 |
|  | Insulin-like growth factor 1 | 342,156 | 1.211 | 1.203 | <0.001 | 3.7 |
|  | Sex hormone-binding globulin | 311,634 | 1.215 | 1.260 | 0.00158 | 0.0 |
|  | Testosterone | 313,755 | 1.232 | 1.236 | 0.442 | 0.0 |
|  | Estradiol | 58,581 | 1.248 | 1.246 | 0.761 | 0.7 |
| ***Prostate cancer*** | | | | | | |
|  | C-reactive protein | 165,697 | 0.944 | 0.948 | 0.0915 | 8.1 |
|  | White blood cell count | 169,494 | 0.949 | 0.952 | 0.339 | 6.2 |
|  | Alanine aminotransferase | 166,081 | 0.946 | 0.958 | 0.0109 | 23.2 |
|  | Gamma-glutamyl transferase | 166,387 | 0.944 | 0.946 | 0.688 | 2.8 |
|  | Insulin-like growth factor 1 | 165,346 | 0.940 | 0.947 | <0.001 | 12.1 |
|  | Sex hormone-binding globulin | 152,502 | 0.931 | 0.910 | <0.001 | 0.0 |
|  | Testosterone | 164,645 | 0.938 | 0.920 | <0.001 | 0.0 |
|  | Estradiol | 14,151 | 0.902 | 0.902 | 0.803 | 0.0 |

***Note.*** Total HR denotes the total association between circadian syndrome and the cancer outcome, and Direct HR denotes the corresponding direct association after accounting for the specified mediator. All mediators were modeled as standardized biomarkers. P values refer to the mediation analysis for the indirect pathway. Proportion mediated is presented as a percentage. ALT, alanine aminotransferase; CRP, C-reactive protein; GGT, gamma-glutamyl transferase; HR, hazard ratio; IGF-1, insulin-like growth factor 1; SHBG, sex hormone-binding globulin; WBC, white blood cell count.

**Table S4. Population-Attributable Fractions of Incident Cancer Associated With Circadian Syndrome for 12 Site-Specific Cancers in the UK Biobank**

| **Cancer Type** | **Baseline Prevalence of CircS (Pe), %** | **Adjusted sdHR** | **PAF, % (95% CI)** |
| --- | --- | --- | --- |
| Endometrial cancer | 20.1 | 1.78 | 13.5 (10.4–16.8) |
| Liver cancer | 22.4 | 1.69 | 13.4 (9.3–17.6) |
| Uterine cancer | 20.1 | 1.70 | 12.4 (9.5–15.4) |
| Kidney cancer | 22.4 | 1.54 | 10.8 (7.9–13.9) |
| Thyroid cancer | 22.4 | 1.41 | 8.4 (2.4–15.1) |
| Esophageal cancer | 22.4 | 1.35 | 7.2 (4.2–10.5) |
| Stomach cancer | 22.4 | 1.29 | 6.1 (2.6–9.8) |
| Pancreatic cancer | 22.4 | 1.28 | 5.9 (3.0–9.0) |
| Lung cancer | 22.4 | 1.24 | 5.2 (3.5–6.9) |
| Bladder cancer | 22.4 | 1.16 | 3.5 (1.6–5.5) |
| Colorectal cancer | 22.4 | 1.09 | 2.0 (0.7–3.4) |
| Breast cancer | 20.1 | 1.06 | 1.2 (0.1–2.3) |

Note. Pe denotes the observed baseline prevalence of circadian syndrome (CircS) in the relevant analytic subcohort; sex-specific cancers (uterine, endometrial, and breast) used the prevalence in the female subcohort, and all other cancers used the prevalence in the full cohort. Adjusted sdHR denotes the subdistribution hazard ratio for incident cancer comparing participants with CircS to those without CircS, estimated from the fully adjusted Fine–Gray competing-risk model. PAF denotes the population-attributable fraction, calculated from Pe and the adjusted sdHR, and represents the proportion of incident cases statistically attributable to CircS under the assumptions of the fitted model rather than the proportion of cases that would be averted by eliminating CircS. The PAF 95% CI was obtained by applying the same formula to the lower and upper 95% confidence limits of the adjusted sdHR, with Pe treated as fixed. Cancer sites are ordered by descending PAF. CI, confidence interval; CircS, circadian syndrome; PAF, population-attributable fraction; sdHR, subdistribution hazard ratio.

**Table S5. Detailed definitions of CircS exposures and incident cancer outcomes**

| **Construct** | **UK Biobank field ID(s) / source** | **Data codes / thresholds** | **Definition** |
| --- | --- | --- | --- |
| ***Exposure assessment*** | | | |
| CircS | Derived study exposure | Present if >=4 of 7 component traits are positive | Binary circadian syndrome exposure defined as the presence of at least 4 of 7 prespecified circadian-metabolic traits. |
| CircS score | Derived study exposure | Range 0-7 | Unweighted sum of the 7 component traits, with higher values indicating a greater burden of circadian-metabolic dysregulation. |
| CircS severity z score | Derived study exposure | Standardized continuous index | Continuous severity index based on standardized waist circumference, log-transformed triglycerides, inverted HDL cholesterol, mean arterial pressure, glycated haemoglobin, sleep deviation from 7.5 hours, and depression-related burden. |
| Central obesity | Fields 48 and 31 | Men: >=102 cm; women: >=88 cm | Defined by sex-specific waist circumference thresholds at baseline. |
| Elevated triglycerides | Field 30870; Field 20003; self-reported history; hospital diagnoses | Triglycerides >=1.7 mmol/L, or lipid-lowering treatment, or prior hyperlipidemia | Defined by elevated baseline triglycerides, lipid-lowering treatment, or prior hyperlipidemia documented from self-report or prebaseline health records. |
| Reduced HDL-C | Field 30760; Field 31; lipid-lowering treatment | Men: <1.0 mmol/L; women: <1.3 mmol/L; or lipid-lowering treatment | Defined by sex-specific low HDL cholesterol thresholds or lipid-lowering treatment. |
| Elevated blood pressure | Fields 4080, 4079, 93, and 94; antihypertensive treatment; self-reported history; hospital diagnoses | SBP >=130 mm Hg, or DBP >=85 mm Hg, or antihypertensive treatment, or prior hypertension | Defined by elevated measured blood pressure, antihypertensive treatment, or prior hypertension documented from self-report or prebaseline health records. |
| Impaired glucose regulation | Fields 30740, 30750, and 74; antidiabetic treatment; self-reported history; hospital diagnoses | Fasting glucose >=5.6 mmol/L among participants fasting >=8 h, or HbA1c >=39 mmol/mol, or antidiabetic treatment, or prior diabetes | Defined by elevated fasting glucose, elevated glycated haemoglobin, antidiabetic treatment, or prior diabetes documented from self-report or prebaseline health records. |
| Short sleep duration | Field 1160 | <6 h/day | Defined by self-reported sleep duration of less than 6 hours per day. |
| Depression | PHQ-9 item fields 20514, 20510, 20517, 20519, 20511, 20507, 20508, 20518, 20513 and repeat fields 29002-29010; Field 20003; Field 20002; Field 41270 | Self-reported history, antidepressant use, prior mood disorder codes, or PHQ-9 score >=5 | Defined at baseline by self-reported depression history, antidepressant use, mood disorder codes, or a PHQ-9 score of at least 5. |
| ***Outcome ascertainment*** | | | |
| Overall incident cancer | Cancer registry ICD-10 (Fields 40006 and 40005) | C00-C97 excluding C44 | All malignant neoplasms captured by ICD-10 codes C00-C97, excluding nonmelanoma skin cancer (C44). |
| Head and neck cancer | Cancer registry ICD-10 (Fields 40006 and 40005) | C00, C01, C02, C03, C04, C05, C06, C07, C08, C09, C10, C11, C12, C13, C14, C32 | Incident head and neck cancer defined using ICD-10 codes C00, C01, C02, C03, C04, C05, C06, C07, C08, C09, C10, C11, C12, C13, C14, C32. Plain-English coding19 meaning: lip; base of tongue; other and unspecified parts of tongue; gum; floor of mouth; palate; other and unspecified parts of mouth; parotid gland; other and unspecified major salivary glands; tonsil; oropharynx; nasopharynx; pyriform sinus; hypopharynx; other and ill-defined sites in the lip, oral cavity and pharynx; larynx. |
| Esophageal cancer | Cancer registry ICD-10 (Fields 40006 and 40005) | C15 | Incident esophageal cancer defined using ICD-10 codes C15. Plain-English coding19 meaning: oesophagus. |
| Stomach cancer | Cancer registry ICD-10 (Fields 40006 and 40005) | C16 | Incident stomach cancer defined using ICD-10 codes C16. Plain-English coding19 meaning: stomach. |
| Colorectal cancer | Cancer registry ICD-10 (Fields 40006 and 40005) | C18, C19, C20 | Incident colorectal cancer defined using ICD-10 codes C18, C19, C20. Plain-English coding19 meaning: colon; rectosigmoid junction; rectum. |
| Liver cancer | Cancer registry ICD-10 (Fields 40006 and 40005) | C22 | Incident liver cancer defined using ICD-10 codes C22. Plain-English coding19 meaning: liver and intrahepatic bile ducts. |
| Pancreatic cancer | Cancer registry ICD-10 (Fields 40006 and 40005) | C25 | Incident pancreatic cancer defined using ICD-10 codes C25. Plain-English coding19 meaning: pancreas. |
| Lung cancer | Cancer registry ICD-10 (Fields 40006 and 40005) | C34 | Incident lung cancer defined using ICD-10 codes C34. Plain-English coding19 meaning: bronchus and lung. |
| Melanoma | Cancer registry ICD-10 (Fields 40006 and 40005) | C43 | Incident melanoma defined using ICD-10 codes C43. Plain-English coding19 meaning: Malignant melanoma of skin. |
| Soft tissue cancer | Cancer registry ICD-10 (Fields 40006 and 40005) | C45, C46, C47, C48, C49 | Incident soft tissue cancer defined using ICD-10 codes C45, C46, C47, C48, C49. Plain-English coding19 meaning: Mesothelioma; Kaposi's sarcoma; peripheral nerves and autonomic nervous system; retroperitoneum and peritoneum; other connective and soft tissue. |
| Breast cancer | Cancer registry ICD-10 (Fields 40006 and 40005) | C50 | Incident breast cancer defined using ICD-10 codes C50. Plain-English coding19 meaning: breast. |
| Cervical cancer | Cancer registry ICD-10 (Fields 40006 and 40005) | C53 | Incident cervical cancer defined using ICD-10 codes C53. Plain-English coding19 meaning: cervix uteri. |
| Endometrial cancer | Cancer registry ICD-10 (Fields 40006 and 40005) | C54 | Incident endometrial cancer defined using ICD-10 codes C54. Plain-English coding19 meaning: corpus uteri. |
| Uterine cancer | Cancer registry ICD-10 (Fields 40006 and 40005) | C53, C54, C55 | Incident uterine cancer defined using ICD-10 codes C53, C54, C55. Plain-English coding19 meaning: cervix uteri; corpus uteri; uterus, part unspecified. |
| Ovarian cancer | Cancer registry ICD-10 (Fields 40006 and 40005) | C56 | Incident ovarian cancer defined using ICD-10 codes C56. Plain-English coding19 meaning: ovary. |
| Prostate cancer | Cancer registry ICD-10 (Fields 40006 and 40005) | C61 | Incident prostate cancer defined using ICD-10 codes C61. Plain-English coding19 meaning: prostate. |
| Kidney cancer | Cancer registry ICD-10 (Fields 40006 and 40005) | C64, C65 | Incident kidney cancer defined using ICD-10 codes C64, C65. Plain-English coding19 meaning: kidney, except renal pelvis; renal pelvis. |
| Bladder cancer | Cancer registry ICD-10 (Fields 40006 and 40005) | C67 | Incident bladder cancer defined using ICD-10 codes C67. Plain-English coding19 meaning: bladder. |
| Brain cancer | Cancer registry ICD-10 (Fields 40006 and 40005) | C71 | Incident brain cancer defined using ICD-10 codes C71. Plain-English coding19 meaning: brain. |
| Thyroid cancer | Cancer registry ICD-10 (Fields 40006 and 40005) | C73 | Incident thyroid cancer defined using ICD-10 codes C73. Plain-English coding19 meaning: thyroid gland. |
| Lymphoma | Cancer registry ICD-10 (Fields 40006 and 40005) | C81, C82, C83, C84, C85, C86, C88 | Incident lymphoma defined using ICD-10 codes C81, C82, C83, C84, C85, C86, C88. Plain-English coding19 meaning: Hodgkin's disease; Follicular [nodular] non-Hodgkin's lymphoma; Diffuse non-Hodgkin's lymphoma; Peripheral and cutaneous T-cell lymphomas; Other and unspecified types of non-Hodgkin's lymphoma; Other specified types of T/NK-cell lymphoma; Malignant immunoproliferative diseases. |
| Non-Hodgkin lymphoma | Cancer registry ICD-10 (Fields 40006 and 40005) | C82, C83, C84, C85 | Incident non-hodgkin lymphoma defined using ICD-10 codes C82, C83, C84, C85. Plain-English coding19 meaning: Follicular [nodular] non-Hodgkin's lymphoma; Diffuse non-Hodgkin's lymphoma; Peripheral and cutaneous T-cell lymphomas; Other and unspecified types of non-Hodgkin's lymphoma. |
| Multiple myeloma | Cancer registry ICD-10 (Fields 40006 and 40005) | C90 | Incident multiple myeloma defined using ICD-10 codes C90. Plain-English coding19 meaning: Multiple myeloma and malignant plasma cell neoplasms. |
| Leukemia | Cancer registry ICD-10 (Fields 40006 and 40005) | C91, C92, C93, C94, C95 | Incident leukemia defined using ICD-10 codes C91, C92, C93, C94, C95. Plain-English coding19 meaning: Lymphoid leukaemia; Myeloid leukaemia; Monocytic leukaemia; Other leukaemias of specified cell type; Leukaemia of unspecified cell type. |

Table note. Abbreviations: CircS, circadian syndrome; DBP, diastolic blood pressure; HbA1c, glycated haemoglobin; HDL-C, high-density lipoprotein cholesterol; ICD-10, International Statistical Classification of Diseases and Related Health Problems, Tenth Revision; IPAQ, International Physical Activity Questionnaire; PHQ-9, 9-item Patient Health Questionnaire; SBP, systolic blood pressure. A CircS component was considered present if any qualifying source indicated a positive finding. Incident cancers were ascertained from national cancer registry records, with follow-up from baseline until first cancer, death, loss to follow-up, or October 31, 2022, whichever occurred first. Sex-specific cancers were evaluated in sex-restricted subcohorts.

**Table S6. Detailed definitions of covariates**

| **Covariate** | **UK Biobank Field ID(s)** | **UK Biobank Field Name(s)** | **Instance(s)** | **Coding** |
| --- | --- | --- | --- | --- |
| ***Primary model*** | | | | |
| Age | 21022 | Age at recruitment | Singular baseline-derived field | Continuous in years |
| Sex | 31 | Sex | Singular baseline field | Male or female |
| Ethnicity | 21000 | Ethnic background | Baseline-focused | Categorical ethnicity grouping derived from self-reported ethnic background |
| Smoking status | 20116 | Smoking status | Instance 0 prioritized | Categorical: never, previous, current |
| Alcohol intake | 20117 | Alcohol drinker status | Instance 0 prioritized | Categorical: never, previous, current |
| Townsend deprivation index | 22189 | Townsend deprivation index at recruitment | Singular baseline-derived field | Continuous score |
| Physical activity | 22032 | IPAQ activity group | Instance 0 prioritized | Categorical IPAQ activity group |
| ***Sensitivity model additions*** | | | | |
| Body mass index (BMI) | 21001 | Body mass index | Instance 0 prioritized | Continuous in kg/m^2 |
| C-reactive protein (CRP) | 30710 | C-reactive protein | Instance 0 prioritized | Continuous in mg/L |

Table note. Abbreviations: BMI, body mass index; CRP, C-reactive protein; IPAQ, International Physical Activity Questionnaire. Covariates were defined at baseline or using baseline-prioritized instances to match the analytic design. Sex was omitted from sex-restricted models. BMI and CRP were included only in sensitivity analyses.
